# The Effect of Adjunctive Antimicrobial Photodynamic Therapy in the Treatment of Peri-Implant Diseases: Systematic Review and Meta-Analysis

**DOI:** 10.3390/dj13120567

**Published:** 2025-12-01

**Authors:** Livia Nastri, Marco Annunziata, Pierluigi Mariani, Agostino Guida, Michele Giuseppe Pio Di Mare, Luigi Guida

**Affiliations:** 1 Multidisciplinary Department of Medical-Surgical and Dental Specialties, University of Campania Luigi Vanvitelli, Via L. De Crecchio, 6, 80138 Naples, Italy; livia.nastri@unicampania.it (L.N.); marco.annunziata@unicampania.it (M.A.);; 2 UOC Odontostomatologia, AORN “A. Cardarelli”, 80131 Naples, Italy

**Keywords:** peri-implant diseases, antimicrobial photodynamic therapy, peri-implant mucositis, peri-implantitis, mechanical debridement

## Abstract

**Background/Objectives**: Peri-implant diseases may occur around osseointegrated implants and lead to implant loss. Treatment strategies focus on infection control with decontamination of implant surfaces/pockets. Mechanical debridement (MD) is necessary to reduce biofilm, although it may have limited effects. Antimicrobial photodynamic therapy (aPDT) has been proposed to increase the potential of MD. The aim of this systematic review and meta-analysis is to evaluate aPDT in adjunct to MD versus MD as a single treatment. **Methods**: An electronic and hand literature search was performed in several databases up to March 2025 to include randomized controlled trials (RCTs). Risk of bias (RoB) was assessed by Cochrane Risk of Bias Tool for RCTs (RoB 2). A meta-analysis was performed with marginal bone level change (MBLc) as the primary outcome, and changes in probing depth (PD) and bleeding on probing (BOP) as secondary outcomes. **Results**: Eleven RCTs (1056 implants, 878 patients) were included. RoB was high: in 3 studies; some concerns: in 4 studies; low: in 4 studies. The included studies showed a high heterogeneity for MD/aPDT protocols and diagnostic criteria of peri-implant diseases. The meta-analysis revealed, for four studies, a significantly higher MBLc for test patients (M∆: 0.29, 95% CI 0.12, 0.46; *p* < 0.001) and a significantly higher BOP change (M∆ 5.59; 95% CI: 1.19, 9.86; *p* = 0.01). No significant difference was found at 6 months in terms of PD change between the test and control groups (M∆ 0.46; 95% CI −0.09, 1.02; *p* = 0.10). High heterogeneity (I^2^ > 85%) for all three outcomes was found. **Conclusions**: High heterogeneity, diagnostic variability, and the low number of included studies increase the need of well-designed RCTs on the topic. Despite no conclusive evidence could be found, adjunctive aPDT showed a promising trend to improve MD results.

## 1. Introduction

Dental implants are rapidly becoming the most common treatment strategy for prosthetic rehabilitation in partially and/or completely edentulous patients. Long- and short-term success of implant-based rehabilitation may be compromised by peri-implant diseases, which may occur around osseointegrated implants and represent one of the most frequent complications that may lead to implant loss [1,2]. Consequently, according to the consensus report from the “11th European Workshop on Periodontal and Peri-implant diseases”, peri-implant diseases are an emerging public health issue [3,4].

Peri-implant diseases include two plaque-associated pathological conditions: peri-implant mucositis and peri-implantitis. Peri-implant mucositis (PIM) is a microbial-induced reversible inflammatory process localized in the peri-implant soft tissues surrounding the implant without signs of bone loss; whereas, peri-implantitis (PI) is an irreversible inflammatory process of both the hard and soft peri-implant tissues. It is characterized by a progressive loss of supporting bone to a point beyond physiological bone remodeling, and, if untreated, it is likely to lead to the failure of the affected implant [5].

Several periodontal pathogens have been shown to colonize peri-implant pockets in PI/PIM: *Porphyromonas gingivalis*, *Prevotella intermedia*, *Prevotella nigrescens*, *Tannerella forsythia*, *Treponema denticola*, and *Fusobacterium nucleatum* [6]. However, some studies show the presence of other microbial species, usually not associated with periodontitis: enteric rods, yeasts, *S. Aureus*, *S. Epidermidis*, *Peptostreptococcus* [7,8,9,10], and others. When comparing the composition of the biofilm surrounding teeth and implants, members of the genera *Staphylococcus* and *Treponema* have been found to be significantly associated with PI but not periodontitis [11].

When compared to periodontitis, histopathological examination of PI lesions reveals progress in direct contact with alveolar bone [12] and a greater proportion of neutrophilic granulocytes and osteoclasts, with the latter found in large numbers in both crestal bone and soft tissue [13]. On these bases, PI is considered more aggressive and less responsive to treatment than periodontitis.

Ideally, treatment of PI/PIM focuses on infection control, obtained by decontamination/detoxification of implant surfaces and pockets, in order to allow for the healing of the affected tissues and stabilize the bone level, minimizing recurrent infections. This can be achieved when the majority of bacterial biofilms and hard deposits are eliminated from the implant surface to obtain a biologically acceptable surface, which will induce wound healing [14]. Various methods have been proposed for the decontamination of implant surfaces and pockets, mechanical debridement (MD) being the most common and studied [15]. Most of the studies on the treatment of PI/PIM by MD reported a reduction in bacterial counts and periodontal pathogen levels in the first 3 months following treatment. However, after 6 months, these improvements tend to vanish because of the recurrence of the infection. Factors such as the shape of the peri-implant bone defects, implant surface characteristics, topography, and presence of threads on the fixtures limit access to the biofilm adhering to the implant, thus favoring infection recurrence [16]. Although nonsurgical MD is the gold standard treatment of periodontal diseases, despite appearing to be beneficial for PIM, it has limited effects on PI, often leading to non-resolution or partial resolution [17,18].

Experimental and clinical studies have essayed a variety of mechanical approaches (including carbon fibers/titanium/plastic curettes, ultrasonic and abrasive air polishing devices) with adjunctive chemical agents (hydrogen peroxide, saline solutions, chlorhexidine (CHX), acid mixtures) [19,20,21] or even Nd: YAG/CO2/Er: YAG lasers [22] and some additional therapies, like locally or systemically delivered antibiotics [23]. Some of these treatments have collateral effects, altering the implant surface, or side effects such as allergies or bacterial resistance. The presence of bacterial complexes with different responses to therapeutic modality also reveals a rationale for combined decontamination methods.

To overcome the aforementioned limitations of PI therapy, innovative clinical approaches have been proposed, and antimicrobial photodynamic therapy (aPDT) has been studied as an adjunct to MD [23]. aPDT induces cellular phototoxicity through an endogenous or exogenous photosensitizer (PS) activated by light at a proper wavelength [24,25]. After light administration, cytotoxicity is usually exerted by the PS itself and/or reactive oxygen species (ROS) created during the process. aPDT involves interactions between various PS, such as methylene blue, toluidine blue (TBO), indocyanine green dye (ICG), phenothiazine chloride, tolonium chloride, etc., and a light source (630 nm to 830 nm according to the PS) in an aerobic environment [26,27]. These interaction releases ROS, causing oxidative damage to microbial cell walls and a photothermal effect, causing microbial cell injury [27].

aPDt has already been employed as an alternative/adjunctive treatment of periodontitis with debatable results. It has been demonstrated that aPDT can be effective in killing in vitro pathogenic bacteria, such as *Porphyromonas gingivalis* or *Fusobacterium nucleatum* [22], while *Aggregatibacter actinomycetemcomitans* (Aa) can be photoinactivated by a red laser in the presence of malachite green [28] or with TBO with a diode laser emitting source [29]. Conversely, a recent RCT showed that the combination of repeated ICG-aPDT and full-mouth ultrasonic debridement (FMUD) provided no benefits except for selective clinical and microbiological improvements compared to FMUD alone [30]. A comparison of conventional debridement with or without adjunctive use of aPDT for chronic periodontitis [29,31] and aggressive ones [32] in humans indicated greater im-provements in clinical parameters in the aPDT group, compared with scaling and root planing (SRP) alone. A recent systematic review on the adjunctive application of aPDT on patients with untreated periodontitis revealed limited and heterogeneous evidence and failed to identify a statistically significant difference between the interventions [33].

An in vitro study on titanium disks [34] assessed the effect of aPDT—with TBO and ICG activated with diode lasers with wavelengths of 635 nm and 808 nm, respec-tively- on Aa biofilm, showing aPDT as an efficient modality for implant surfaces de-contamination. Moreover, Dortubudak et al. [35] and Bombeccari et al. [36] showed promising results in reducing total bacterial counts and specific periodontal pathogen levels in patients with peri-implantitis treated with aPDT. Furthermore, the use of aPDT as a potential alternative to local antibiotics has been evaluated in clinical studies comparing mechanical nonsurgical treatment of PI followed by either the use of local antibiotics (e.g., minocycline) or application of aPDT. The results at six months and at one year have failed to reveal statistically or clinically significant differences between the two treatment protocols, suggesting aPDT as an alternative to local antibiotics, as it failed to show any harmful potential [37,38]. Some controlled clinical trials have been published on aPDT in adjunct to conventional MD compared to MD alone in the treatment of PI, with conflicting results.

The clinical usefulness of this therapeutic approach is thus still a matter of debate. The aim of this systematic review is to evaluate the clinical effect of aPDT as an adjunct to mechanical debridement in cases of PIM and PI compared to mechanical debridement alone.

## 2. Materials and Methods

This systematic review was performed in accordance with the statement of preferred reporting items for systematic reviews and meta-analyses (PRISMA) [39]. (Prisma Checklist in Table S1).

A focused question was formulated in the PICO format: “Does aPDT, in adjunct to MD, reach significantly better therapeutic results than MD alone in patients with peri-implant disease?”

Population (P), Intervention (I), Comparison (C), and Outcomes (O)—PICO

- Population: Patients diagnosed with peri-implant disease (PIM or PI)
- Intervention: Use of aPDT as an adjunct to MD (with or without flap elevation)
- Comparison: Use of MD alone versus MD with adjunctive aPDT
- Outcome: Evaluation of clinical and/or radiographic effects.

### 2.1. Search Strategy

Research on different medical-scientific databases (PubMed, Embase, Cochrane Database, ResearchGate, Google Scholar, ClinicalTrials.gov, OpenGray) was performed, limiting the date to 1 March 2025.

We used the following terms as keywords: (Antimicrobial photodynamic therapy OR photodynamic therapy OR laser therapy OR laser OR antimicrobical laser therapy OR photochemotherapy OR Photodynamic Therapies OR aPDT OR PDT OR photody-namic therapy OR photo-bio-modulation OR diode OR photosensitizer OR Phenothi-azine chloride OR Methylene blue OR Toluidine Blue OR ICG OR indocyanine green) AND (perimplant disease OR peri implant disease OR perimplant mucositis OR per-implant mucositis OR peri-implant mucosal inflammation OR perimplantitis OR peri implantitis OR peri-implant bone loss OR peri-implant defect OR peri-implant tissue loss OR dental implants) AND (RCT OR randomized controlled trial).

### 2.2. Eligibility Criteria

Studies meeting the following inclusion criteria were included:

- Randomized Controlled Trials in humans comparing MD + aPDT in the test group and MD alone in the control group (with or without flap elevation) for the treatment of peri-implant diseases;
- Defined diagnostic criteria of peri-implant disease (mucositis or peri-implantitis);
- Studies written in English;
- Studies involving 10 or more patients with one or more implants each;
- Studies with a minimum follow-up period of at least six months after treatment;
- Studies that utilized any PS solution (at any dose) combined with any light technology and wavelength;
- Studies reporting at least one of the following parameters as outcome variables: probing depth, bleeding on probing, and marginal bone level.

The exclusion criteria were:

- In vitro studies, animal studies, case reports and case series, non-randomized clinical trials, prospective and retrospective observational studies, pilot studies, narrative literature reviews, letters to the editor, and congress abstracts or posters;
- Studies where subjects assumed antibiotic therapy in the last three months prior to RCT enrolment;
- Studies using additional antibiotics or other adjunctive therapies in the test or control group;
- Studies in which aPDT was performed after surgery.

### 2.3. Study Selection and Data Extraction

All articles were initially screened by two independent reviewers (LN and MA) based on titles and abstracts, and imported into a reference manager to remove any duplicates. The second stage of screening involved a full-text reading using a predetermined data extraction form to confirm the eligibility of each study based on the aforementioned inclusion and exclusion criteria. In particular, the characteristics of all included studies were collated, including population, intervention, comparator, outcomes, and study design. These data were tabulated in evidence tables and then compared against the predefined eligibility criteria for each planned synthesis. Any disagreements were resolved via discussion between the two reviewers. The data on patient characteristics, treatment covariates, and clinical outcomes for each available follow-up were independently extracted from all the eligible studies by two reviewers.

A change in marginal bone level (MBL) between the baseline and a minimum 6-month follow-up was chosen as the primary clinical outcome; changes in pocket depth (PD) and bleeding on probing (BOP) were chosen as secondary outcomes. If not directly reported, the outcomes were calculated starting from the baseline/follow-up mean values and standard deviations provided by the articles. If both mesial and distal MBL values were provided, only mesial values were used for calculations.

### 2.4. Risk of Bias (Quality) Assessment

The quality of the included studies was assessed by two independent, calibrated examiners (LN and MA) using the Cochrane Risk of Bias Tool for RCTs (RoB 2) (updated on 22 August 2019) [40]. For each RCT, five domains were considered: (1) bias arising from the randomization process; (2) bias due to deviations from intended interventions; (3) bias due to missing outcome data; (4) bias in the measurement of the outcome; (5) bias in the selection of the reported result. Each domain was ranked as low, uncertain, or high risk. The overall risk of bias of the included studies was categorized as low if all criteria were judged at low risk; as high if one or more criteria were deemed high risk; and at moderate risk if one or more criteria were unclear and none were at high risk.

### 2.5. Meta-Analysis

A meta-analysis was performed with MBL change (MBLc) as the primary outcome, and changes in PD and BOP as secondary outcomes. Negative and positive MBLc values were used to indicate bone loss and bone gain, respectively. Effect sizes were displayed as mean difference (subsequently referred to as M∆), with 95% confidence intervals. Forest plots were created to illustrate the effects of the different studies and global estimation. Potential sources of heterogeneity were explored through subgroup analyses and, where appropriate, meta-regression models. Sensitivity analyses were performed by sequentially excluding individual studies and by applying alternative statistical models to evaluate the robustness of the pooled estimates.

RevMan 5 software (Review Manager, version 5.4, the Cochrane Collaboration, 2020, London, UK) was used to perform all analyses. Statistical significance was defined as a *p*-value < 0.05. The study-specific estimates were pooled with the random-effects models if heterogeneity across trials, tested with the Chi^2^ (Cochran Q) test (*p* < 0.1) and I^2^ statistics > 50%, proved to be high [41]. If the number of included clinical trials was sufficient (ten trials minimum); thus, the visual inspection of funnel plot results was meaningful, and the results were considered as a tool for the assessment of publication bias.

## 3. Results

### 3.1. Study Selection

**Figure 1:**
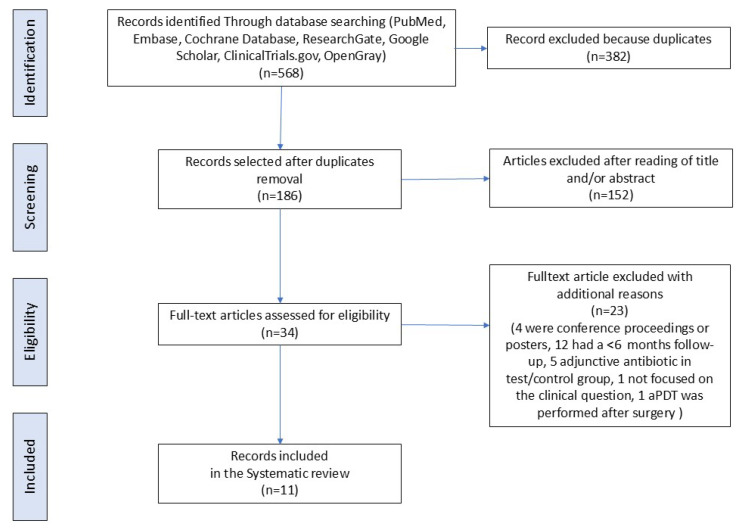
Flow diagram (PRISMA format) of the screening and selection process.

The search strategy identified a total of 568 articles. After the exclusion of duplicates, 186 articles underwent title and/or abstract examination, identifying 34 potentially eligible studies. The screening process is summarized in Supplementary Table S2. After full-text evaluation, 23 failed to meet the inclusion criteria: in 5 of them [37,38,42,43,44], the test group also received antibiotic or antiseptic or adjunctive therapies; in 12 studies [45,46,47,48,49,50,51,52,53,54,55,56], the follow-up was less than 6 months; in 1 paper [57], aPDT was performed after surgery; 1 study [58] was excluded because the trial was not focused on our clinical question; and 4 were excluded because they were either congress proceedings or a poster [59,60,61,62] (Figure 1).

Hence, 11 RCTs [36,63,64,65,66,67,68,69,70,71,72], for a total of 1056 implants and 878 patients, were included in this systematic review (Figure 1).

The details of each included study are reported in Table 1. The criteria for diagnosing peri-implant diseases were different among the studies. All the studies evaluated probing depth, establishing a threshold for the definition of the disease as 4 mm of pocket in six studies [36,64,67,68,69,72], whereas a threshold of 6 mm was set in three studies [63,65,68]. MBL was evaluated with periapical or bitewing radiographs, with a threshold of 2 mm in one study [67], 3 mm in four others [63,70,71,72], and 3 threads with a progressive pattern in another study [36]. In total, 8 out of 11 studies evaluated BOP as a diagnostic criterion [36,63,64,65,67,68,69,72], one used the sulcular bleeding index (SBI) [66], two referred in general terms to inflammation in the soft tissues [70,71], and five also evaluated the presence of exudation [36,63,66,69,70].

Most of the studies (7 out of 11) considered one implant per patient [64,65,66,67,70,71,72], whereas the others evaluated one or more implants [36,63,68,69]. With regards to the studied cohorts, two studies compared the results in non-smokers vs. smoking patients [68,72], three studies [63,65,67] compared results in healthy vs. diabetic patients, and one [64] compared healthy vs. prediabetic patients.

One study considered several systemic diseases as exclusion criteria, while in Esposito et al. [70], systemic conditions were considered for the exclusion of patients only in the case of an impediment to treatment.

Five RCTs [36,63,68,71,72] provided information about the involved implants, such as implant surface, prosthetic connection, and adequacy of implant crown. In none of the studies, the removal of the prosthetic parts was suggested to facilitate access to the contaminated implant surface(s) [73,74,75,76].

The included studies compared MD to adjunctive aPDT; nevertheless, MD was performed in different modalities among the involved studies. In total, 6 out of 11 studies described the use of an ultrasonic device for scaling [63,64,65,67,69,70], 3 of them used plastic curettes on the implant surface [36,71,72]. In two studies [69,70], different instruments (hand instruments, glycine powder) were used together with the ultrasonic debridement on the implant surface. Another study [66] employed a glycine powder air-polishing alone on the implant surface, whereas in one case [68], the method for mechanical debridement was not declared.

Main settings of the aPDT used in the test groups are summarized in Table 2. In all studies, aPDT was carried out with a diode light at different wavelengths. A wavelength of 660 nm was employed in 54.54% [64,65,67,68,71,72] of the studies; 810 nm in 18.18% [36,63]; 630 nm in 9.09% [70]; 635 nm in 9.09% [66]; 670 nm in 9.9% [69].

As PS, indocyanine green was used by 9.09% of the studies [63]; 27.8% used toluidine blue [36,66,70]; 18.18% used methylene blue [71,72]; 45.45% [64,65,67,68,69] used Phenothiazine chloride.

aPDT was generally performed once after MD. One study reported that aPDT was repeated if necessary [70]; another study [63] repeated aPDT after 7, 17, and 27 days. In one study [71], the patients were divided into groups, one receiving a single application of aPDT and two groups receiving multiple applications. Two studies involved the raising of a flap [36,70].

Clinical and radiographic outcomes, with conclusions from each study, are summarized in Table 3. The minimum follow-up interval requested by our inclusion criteria was 6 months. Three studies [67,68,70] reported 12-month follow-up data, and one reported 9-month follow-up [71]. All studies showed intermediate evaluations of the recorded clinical parameters (e.g., 4, 6, and 12 weeks).

Meta-analyses for MBL, PD, and BOP changes were performed, comparing MD + aPDT versus MD alone, at 6 and 12 months, without any surgical procedure [63,65,67,70,71,72]. The study by AlHarthy et al. [71] reported data from the 9-month follow-up evaluations only; thus, it was excluded from the meta-analysis and only qualitatively evaluated due to the impossibility of retrieving the 6-month data to be compared to the other studies.

Of the included studies, MBLc was evaluated in six out of eleven, testing aPDT with [70] or without [63,65,67,68,72] flap elevation. Among these papers, three studies show no differences between the test and control groups over a 6-month period [67,72] or over a 12-month period [68], and two showed differences at the 6-month observation with a substantial reduction in bone loss in the test groups [63,65].

Three studies did not show the numerical values for PD [64,67,68]. Another study did not provide its standard deviation values [69]. For this reason, they were excluded from the quantitative meta-analysis of probing depth variation. In the other four studies (6 data sets), initial PD varied from 4.5 mm to 6.8 mm in the test groups and from 4.5 mm to 6.9 mm in the control groups. After treatment with MD only, PD decreased to a range of 2.6 mm to 5.5 mm, while it reduced to a range from 2 mm to 5.14 mm in the aPDT groups.

Reduction in BOP was reported in both control and test groups, with heterogeneous results. Javed et al. [68] was excluded from the BOP meta-analysis, as the study did not provide numerical values for this clinical outcome; Romeo et al. [69] was excluded because standard deviation values were not provided; Wang et al. [66] was not included as the study used the sulcular bleeding index to evaluate peri-implant bleeding.

Only two studies mentioned some additional deplaquing as an intermediate treatment from the baseline to the end of the study (Esposito et al. [70] and Romeo et al. [69]), while the others did not declare any professional plaque control during the observational time. In four studies [65,69,70,72], authors specified that they gave indications regarding oral hygiene to the patients, mostly consisting of toothbrushing and flossing. Labban et al. [63] reported 75% of their patients toothbrushed once a day, and the other 25% brushed twice a day. No information was given about flossing/interdental hygiene.

Finally, it is worth noting that most of the included studies [63,64,65,67,68,71,72] were published by a single research group.

### 3.2. Risk of Bias and Power of Analysis

**Figure 2:**
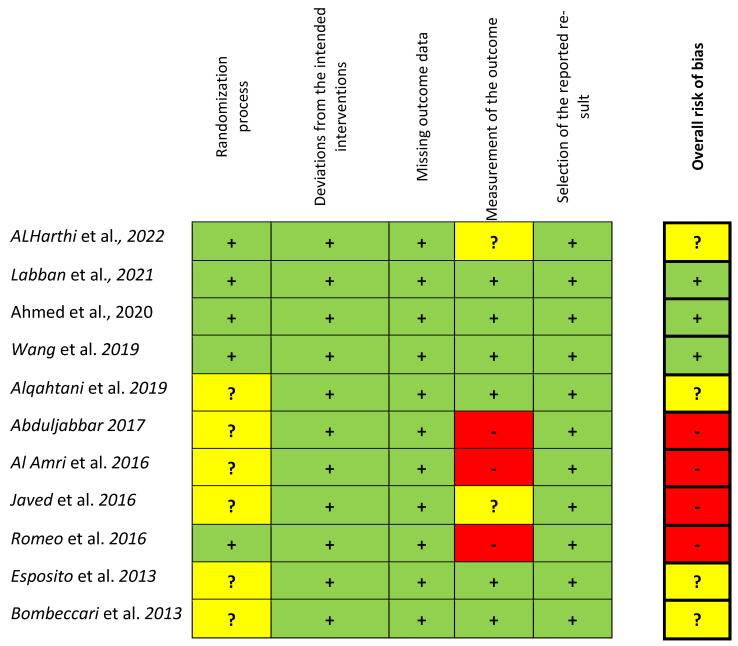
Risk of bias for the included studies according to the Cochrane Risk of Bias Tool for RCTs (RoB 2) based on the consensual answers across investigators. (green colour = low ROB, yellow = unclear (some concerns) ROB. Red = High ROB) [36,63,64,65,66,67,68,69,70,71,72].

The overall risk of bias, evaluated according to the Cochrane Risk of Bias Tool for RCTs (RoB 2) and based on the consensual answers across investigators, for each included study, is shown in Figure 2. Three studies [63,65,66] fulfilled all criteria with a low risk of bias. Four studies [36,70,71,72] showed at least an unclear (some concerns) risk of bias, mainly concerning the randomization process (no details about the allocation concealment) and measurements of the outcome. Four studies [64,67,68,69] were considered at high risk due to possible bias in the outcome measurements. Seven studies [63,65,66,68,70,71,72] reported information on sample size calculation and power analysis, and one study [70] was underpowered.

### 3.3. Meta-Analysis

**Figure 3:**
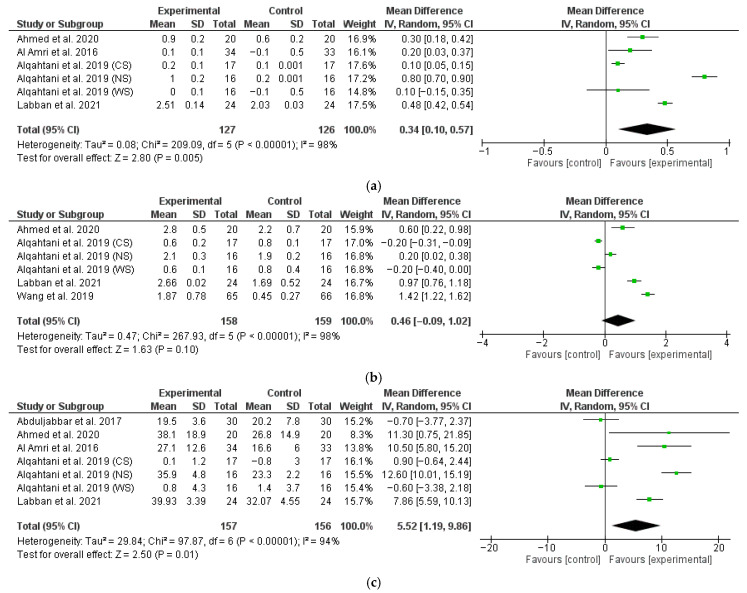
Six-month follow-up forest plots of MBL (**a**), PD (**b**), and BOP (**c**) changes [63,64,65,67,72].

The data from therapy outcomes and follow-up were analyzed separately. Javed et al. did not report rough data regarding MBL, PD, and BOP at either the 6- or 12-month follow-up, so their study could not be included in the meta-analysis. Romeo et al. [69] did not report standard deviation for the outcomes; thus, we were not able to meta-analyze their data. Forest plots of the MBL, PD, and BOP variation comparing experimental vs. control non-surgical treatments at the 6-month follow-up are shown in Figure 3. The estimates were pooled using a random effect model due to the high heterogeneity found (I^2^ = 98%, *p* < 0.001, I^2^ = 98%, *p* < 0.001, I^2^ = 94%, *p* < 0.001 for MBL, PD, and BOP change, respectively). For one study [70], the data on smokers, non-smokers, and former smokers were analyzed as three separate data sets.

The meta-analysis conducted on four studies (six data sets) revealed a significantly higher marginal bone gain for test patients with a M∆ of 0.34 (95% CI: 0.10, 0.57; *p* < 0.01) (Figure 3a).

The meta-analysis conducted on four studies (six data sets) revealed no significant difference in terms of PD change between test and control patients with a M∆ of 0.46 (95% CI: −0.09, 1.02; *p* = 0.10) (Figure 3b).

The meta-analysis conducted on five studies (seven data sets) revealed a significantly higher BOP change for test patients with a M∆ of 5.52 (95% CI: 1.19, 9.86; *p* = 0.01) (Figure 3c).

**Figure 4:**
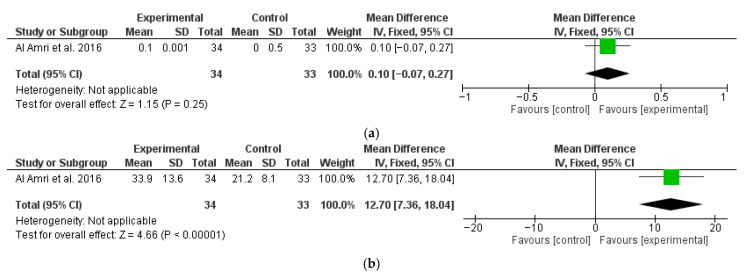
Twelve-month follow-up changes in MBL (**a**) and BOP (**b**) [67].

Another study [67] also reported 12-month follow-up data regarding MBL (Figure 4a) and BOP (Figure 4b), which were analyzed separately. A significantly higher change in favor of the test group was found for BOP, with a M∆ of 12.7 (95% CI: 7.36, 18.04; *p* < 0.01), while MBL did not show statistically significant differences between the test and control groups, with a M∆ of 0.10 mm (95% CI: −0.07, 0.27; *p* = 0.25). Esposito et al. [70], even though it had 12-month results, could not be included in the meta-analysis as it was not possible to separate the data between surgical treatment and non-surgical treatments.

**Figure 5:**
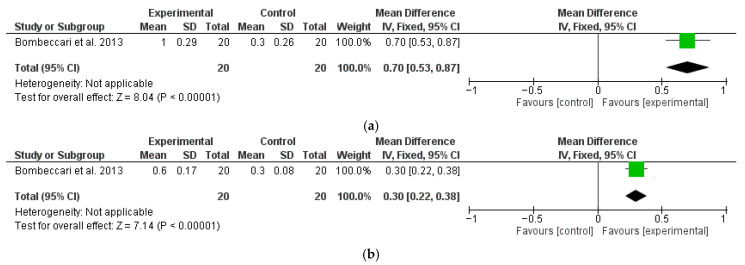
Six-month follow-up changes in PD (**a**) and BOP (**b**) after PDT and MD with surgical therapy (OFD) [35].

Only one of the included studies performed a 6-month follow-up analysis of PD and BOP after surgical therapy, with or without aPDT (Figure 5a and Figure 5b, respectively). The analysis demonstrated a significantly higher reduction in PD with a M∆ of 0.7 (95% CI: 0.53, 0.86; *p* < 0.01) and BOP with a M∆ of 0.3 (95% CI: 0.22, 0.38; *p* < 0.01) for the test groups compared to the control.

Neither sensitivity analysis nor publication bias assessment through a funnel plot was conducted, owing to the limited number of included studies.

## 4. Discussion

As the number of implant-supported dental prostheses is increasing worldwide, peri-implant diseases are a major concern for oral health, and finding a gold standard treatment is a pressing request from clinicians and patients. aPDT has been proposed as a support therapy for MD, which may reduce plaque and consequent inflammation. According to our systematic review and meta-analysis, aPDT in adjunct to MD showed a significant decrease in the peri-implant PD and BOP at 6 months after treatment when compared to MD alone. These clinical outcomes may be accompanied by a reduction in the radiographic peri-implant bone loss after treatment [63,65,67,68,70,72].

Despite these results, some methodological aspects of the included studies need to be addressed. The included studies show a very high heterogeneity, both in treatment protocols and in diagnostic criteria. We decided to review studies based on the treatment of both PIM and PI, in order to evaluate treatment modalities that may be helpful both in initial and advanced clinical conditions. In the 3rd EAO consensus conference on Peri-implant tissue destruction, it was stated that “the continuum from peri-implant mucositis to peri-implantitis is difficult to determine [73]. It is important to treat early signs of inflammation to prevent or limit marginal bone loss” [77]. As a matter of fact, we have not found among the reviewed studies a univocal diagnostic criterion to define mucositis or peri-implantitis; similarly, the presence of bone loss was not univocally considered by every paper as pathognomonic for PI [36,64,66,67,69]. None of the included studies used the definition of peri-implantitis as described by Berglundh et al. [78] or as previously described by Sanz et al. [74].

In 6 out of 11 studies, 6-month follow-up data showed that PD values were significantly reduced when adjunctive aPDT was performed [36,63,65,66,67,68]. Intergroup comparison showed no significant PD changes in the remaining four studies [64,69,70,72]. In the study by Romeo et al. [69], despite the pronounced arithmetic changes from baseline, the authors did not provide the standard deviation or any statistical analysis comparing the outcomes between MD with adjunctive aPDT versus MD alone [69], and was thus excluded from meta-analysis.

In five studies [36,63,65,67,68], BOP, a clinical marker of inflammation, was significantly reduced in the test group after 6 months. One study that evaluated SBI found that it was significantly reduced after treatment with adjunctive aPDT [66]. At the 12-month interval, Javed et al. [68] no longer found a significant difference between test and control treatments; however, the raw data were not available, so they could not be included in the meta-analysis.

MBLc was quantitatively evaluated by six studies [63,65,67,68,70,72]. Two studies revealed both an intragroup and intergroup statistically significant difference at baseline and follow-up [63,65] while the others did not find any intra- or intergroup statistical difference.

Looking at the meta-analyzed data, a significantly higher marginal bone gain and BOP reduction were found in the aPDT groups at 6 months, while PD reduction did not show a significant intergroup difference. These findings, however, need to be considered with caution, due to the reduced number of studies included in the analysis, small sample size, and high heterogeneity among studies.

Furthermore, at the 12-month follow-up, only one study was meta-analyzed, showing a significantly greater BOP reduction at 12 months in the test group [67]. Such promising results need to be considered cautiously, since they derive from a single study.

In this view, despite the favorable outcomes that some studies show, some concerns have to be highlighted.

MD, as a control treatment, was performed in different modalities among the included studies, and the expected outcomes of the described modalities could be debated. In the study by Javed et al. [68], details of MD were not provided, while in four studies [63,64,65,67], it consisted of ultrasonic debridement of the contaminated implant surface. The latter is not a method considered by the scientific literature as a sufficient treatment for peri-implant disease, despite being considered acceptable for periodontal diseases. In the study of Wang et al. [66], MD consisted of air polishing with glycine, with no other decontaminating phase. It is worth noting that there is no gold standard MD modality for peri-implant diseases [11]. Moreover, the MD outcome may be less predictable for deeper defects. Notably, in the study of Esposito et al. [70], which included very deep defects, the adjunctive aPDT did not enhance the clinical outcomes, perhaps indicating that, since oxygen is crucial for the efficacy of aPDT, potential anaerobiosis of deeper pockets may impair photodynamic reactions.

The protocol for aPDT treatment was not univocal. Not only did the PS and wavelengths differ among the studies, but so did the settings of power and frequency. It is noteworthy that the frequency and duration of aPDT (1–4 times and 10 s–100 s, respectively) considerably varied among the studies, as well as the fiber diameter, with different effects on the increase in temperature and possibly influencing efficacy [79].

As previously underlined, lack of long-term follow-up did not allow for an evaluation of the stability of clinical outcomes obtained with aPDT. Three [67,68,70] out of eleven studies presented only a 12-month follow-up, crucially impairing the evaluation of the stability of the clinical outcomes. Interestingly, two of the studies [68,70] did not show an advantage in adjunctive aPDT when presenting the 12-month outcomes. Moreover, with the exception of Romeo et al. [69] and Esposito et al. [70], most of the included studies did not provide any information about supportive periodontal and peri-implant therapy during the follow-up. Only in seven studies [63,65,66,69,70,72] was plaque control mentioned. Oral hygiene instructions, often limited to toothbrushing and flossing, were given in four studies [65,69,70,72], without a difference among implant single crowns, bridges, or complete arches, conditions that may affect the patient’s ability to perform proper plaque control. Moreover, the outcome and progression of PIM/PI are influenced by many factors such as the threads and surface of the fixture, prosthetic connection, and restorative outline. These factors may influence both access to the peri-implant pocket and home hygiene maneuvers performed by the patient [73]. Only five studies gave information (incomplete) on the affected implants [36,63,68,71,72]. This aspect is relevant, considering that the development of appropriate protocols for the prevention of infectious implant disorders through efficient oral hygiene maintenance is crucial. It has been demonstrated how patient-centered motivational models and adjunctive precise home-care protocols may be helpful in preventing or managing peri-implant diseases [80].

When put together, the inconsistency of aPDT protocols, differences in the methodological assessment of peri-implant diseases, high heterogeneity in diagnostic criteria, lack of validation of mechanical treatments used as a control, and short-term follow-ups available limit the results of this systematic review and meta-analysis. This is in agreement with the systematic reviews that were previously published and that used different inclusion criteria, highlighting similar limits and providing similar minimal evidence of additional benefits with aPDT [81,82,83]. In the systematic review by Chala et al. [81], the two included studies on aPDT focused on non-surgical and surgical approaches, respectively. The authors’ findings stated that there were no results to be confirmed, since there was a lack of clinical measurements in the first study, and there was no statistical analysis in the second. In another recent systematic review, the included studies on the adjunctive effect of aPDT for peri-implantitis treatment were limited to two, with a conclusion that both therapies failed at improving clinical outcomes significantly [82]. In a more recent systematic review [83] focusing on papers on patients with a smoking habit, four studies were included. The authors concluded that the PD and plaque index decreased significantly with the use of adjunctive aPDT. The authors also suggested performing more studies on smokers with multiple sessions of aPDT to validate its effects. Interestingly, in our analysis, favorable outcomes were shown by Labban et al. [63], where aPDT was performed more than once, and confirmed by the outcomes from AlHarthy et al. [71], where the groups with a repeated application of aPDT showed an improvement in PD and BOP, even if no changes in marginal bone level were noted. Unfortunately, this protocol has not been adopted by other included studies, and it could not be validated. The repeated application of aPDT could be an interesting aspect for further investigation.

Future RCTs on aPDT should focus on antibacterial/clinical potential advantages and cost risk analysis, both in terms of overcoming the limits of implant MD and grasping the precise aPDT PS/wavelength parameters to be used in different conditions.

## 5. Conclusions

High methodological heterogeneity and a low number of included studies limit the efficacy of this systematic review and meta-analysis on adjunctive aPDT for peri-implant diseases. Thorough RCTs are needed with standardization in terms of peri-implant disease definition, inclusion criteria, home hygiene instructions, and aPDT parameters (photosensitizer, wavelength, energy output, fiber diameter, time and frequency of application, etc.). Short-term follow-up is another major issue with the present literature, which hopefully can be overcome in the future.

Consequently, no recommendation could be extrapolated from the present literature; still, the scant evidence available suggests a certain trend in the short-term in favor of aPDT as an adjunctive therapy for MD for peri-implant diseases, making it worthy for further and thorough investigation.

## Figures and Tables

**Table 1:** Main characteristic of the selected studies.

	First Author, Publication Year	Diagnoses of Peri-Implant Diseases	Type of Study	Number of Implants	Mean Age Years (±SD or Min-Max)	Number of Subjects	aPDT + Surgical/ Non-Surgical Therapy	Follow-Up	Groups	Main Features of Implants or Prosthetic Connection
1	ALHarthi et al., 2022 [71]	MBL > 3 mm (from 2 mm below the implant–abutment junction) in bitewing radiographs	RCT	88	G-I: 59.2 (±5.3) G-II: 60.5 (±2.8) G-III:59.6 (±3.1) G-IV:58.7 (±0.8)	88 (22 + 22 + 22 + 22)	Non-Surgical	9 months	Group-I: MD Group-II: MD + single aPDT Group-III: MD + aPDT at baseline and 3 months Group-IV: MD + aPDT at baseline, at 3 months, and at 6 months	Implant length ≥ 11 ≤ 14 mm; implant diameter ≥ 4.1 ≤ 4.8 mm Platform-switched Delayed loaded In function from 8.5 (±0.2) to 9.2 (±0.2) years
2	Labban et al., 2021 [63]	Assessment of PI, BOP, and PD at six sites, suppuration. CBL on periapical radiographs.	RCT	64 (TG: 35+ CG: 29)	TG: 47.8 (±7.2) CG: 50.4 (±9.3)	48 (TG: 24 + CG: 24)	Non-Surgical	6 months	TG: ICG-PDT + PIMD CG: PIMD alone All patients type 2 DM	Implant design: Platform-switched with moderately rough surfaces Implant in place for about 9 years Position of implants (maxilla/mandible): TG 10/25 and CG 12/17
3	Ahmed et al., 2020 [65]	PD ≥ 6 mm on at least one implant site; ≥3 mm MBL change	RCT	40 (One for subject)	G1: 48.9 (±4.5) G2: 51.4 (±6.7) G3: 50.7 (±5.9)	60 (20 + 20 + 20)	Non-Surgical	6 months	G1 (20): MD + single session of aPDT G2 (20)Not Considered G3 (20): MD alone All patients with type 2 Diabetes Mellitus (DM)	NA
4	Alqahtani et al., 2019 [72]	Assessment of PI, BOP, and PD and crestal bone level on digital bitewing radiographs.	RCT	98 (One for subject)	CS Group: 52.3 (±2.2) WS Group: 55.6 (±1.6) NS Group: 54.2 (±2.2)	98 (TG: 49 + CG: 49)	Non-Surgical	6 months	Test group (49): MD + aPDT; Cigarette-smokers (17) + Waterpipe-smokers (16) + Never-smokers (16) Control group (49): MD alone; Cigarette-smokers (17) + Waterpipe-smokers (16) + Never-smokers (16)	Implant design: Platform-switched with moderately rough surfaces The length and diameter of the implants placed ranged between 11 and 14 mm and 4.1 and 4.8 mm, respectively Implant in place about 5 years Position of implants: implant placed in either jaws in the premolar or molar region
5	Wang et al., 2019 [66]	Evaluation of inflammatory symptoms, bone loss by X-ray examination, BOP, and suppuration.	RCT	131 (One for subject)	43.4 (±11.5)	131	Non-Surgical	6 months	Test group (65): full-mouth cleansing for 2 weeks (+undergo periodontal pocket cleansing for patients with periodontitis) + underwent subgingival sand-blast using glycine powder + PDT; Control group (66): full-mouth cleansing for 2 weeks (+undergo periodontal pocket cleansing for patients with periodontitis) +underwent subgingival sand-blast using glycine powder + saline solution.	NA
6	Abduljabbar 2017 [64]	BOP+ and PD ≥ 4 mm at 6 sites per implant and presented as mean percentages per individual.	RCT	60 (One for subject)	TG: 50.6 (±1.4) CG: 51.4 (±0.6)	60 Prediabetic patients (HbA1c ≥ 5.7% < 6.4%)	Non-Surgical	6 months	Test group (30): MD + aPDT Control group (30): MD alone Patients with medically diagnosed prediabetes (hemoglobin A1c [HbA1c] levels ≥ 5.7% to 6.4%)	NA
7	Al Amri et al., 2016 [67]	BOP+ in >30% sites and/or MBL of >2 mm.	RCT	67 (One for subject)	TG: 53.6 (±9.5) CG: 51.4 (±3.7)	67 All patients with type 2 DM	Non-Surgical	12 months	Test group (34): MD + aPDT Control group (33): MD alone All patients are type 2 DM	NA
8	Javed et al., 2016 [68]	BOP in at least 30% sites and PD of at least 4 mm.	RCT	245 (125 = in cigarette smoker patients; 124 = in non-smoker patients)	40 years (26–54 years)	166 (84 = CS; 82 = non-smokers)	Non-Surgical	12 months	Test group (41 cigarette smokers + 40 non-smokers): SRP + MD + aPDT; Control group (43 cigarette smokers + 42 non-smokers): SRP + MD	Implant design: Platform-switched with moderately rough surfaces The length and diameter of the implants placed in the patients’ groups ranged between 11 and 14 mm and 3.5 and 4.1 mm, respectively Delayed-loaded
9	Romeo et al., 2016 [69]	PD ≥ 4 mm, BOP and suppuration.	RCT	123	34–68	40	Non-Surgical	6 months	Test group (63 implants): Ultrasonic debridement + PDT; Control group (59 implants): Ultrasonic debridement	NA
10	Esposito et al., 2013 [70]	MBL of >3 mm, pus exudation and/or soft tissue swelling and/or soft tissue redness.	RCT	30 (One for subject)	25–80	80	MBL ≤ 5 mm: Non-surgical MBL > 5 mm: Surgical	1 year	Test group (15): MD or OFD (depending on the amount of MBL) + LAD therapy Control group (15): MD or OFD (depending on the amount of MBL)	NA
11	Bombeccari et al., 2013 [36]	PD > 5 mm, BOP and/or exudation. Radiographic signs of progressive bone loss > 3 threads.	RCT	40	46 (33–64)	40	Surgical	6 months	Test group (20): OFD + PDT; Control group (20): OFD alone	Moderately rough surface implants

LEGEND: RCT = randomized controlled trial; SD = standard deviation; aPDT = antimicrobial photodynamic therapy; PD = probing depth; BOP = bleeding on probing; PI = plaque index; MBL = marginal bone loss; DM = diabetes mellitus; TG = test group; CG = control group; PS = platform-switched connection; CS = cigarette smokers; WS = waterpipe smokers; NS = never smokers; MD = mechanical debridement; OFD = open flap debridement; NA = not assessed.

**Table 2:** Main settings and protocols of the photodynamic therapy applied in each included study.

	First Author, Publication Year	Type of Light (Manufacter)	Wavelength	Photosensitizer (Concentration)/Pre-Irradiation Time)	Application of Photosensitizer	Removal of Photosensitizer Before Irradiation	Adjunctive PDT	Irradiation Time	Irradiation Location	Energy (J)	Dose (J cm^2^)	Output Power (mW)	Irradiance (mw/cm^2^)	Fiber and Spot
1	ALHarthi et al., 2022 [71]	Diode laser	660 nm	0.005% of Methylene blue/15 s	Applied into the deepest buccal peri-implant pocket and left in place	NA	In groups III (at 3 months) and IV (3 and 6 months)	60 s per site	NA	3 J	0.0125 J/cm^2^	180 mW (continuous ave)	NA	600 μm
2	Labban et al., 2021 [63]	Diode laser (A.R.C. laser GmbH, Nurnberg, Germany)	810 nm	1 mg/mL ICG solution	Applied until the bottom of the peri-implant pocket using a 1 mL syringe	NA	After 7, 17, and 27 days	50 s	Starting from the papilla for 30 s, followed by the insertion inside the peri-implant pocket depth for 10 s from both buccal and lingual sides, moving to coronal direction	4 J	NA	200 mW (continuous mode)	NA	NA
3	Ahmed et al., 2020 [65]	Diode laser (HELBO TheraLite Laser, HELBO 3D Pocket Probe; Photodynamic Systems GmbH, Senden, Germany)	660 nm	Phenothiazine chloride, 0.005% concentration/120 s	Transferred into the peri-implant pockets via a blunt needle until the base gradually.	NA	NO	10 s at each site	Peri-implant area	NA	NA	150 mW	1.1 W/cm^2^	NA
4	Alqahtani et al., 2019 [72]	Diode laser	660 nm	0.005% of Methylene blue/10 s	Applied into the deepest buccal peri-implant pocket and left in place	NA	NO	60 s per site	NA	3 J	0.0125 J/cm^2^	150 mW	NA	600 µm
5	Wang et al., 2019 [66]	Light-emitting diode	635 nm	0.5 mL of Toulidine Blue (10 mg/mL)/3 min	Injected from the pocket bottom around the implant	Sterile water	NO	10 s per site	NA	NA	NA	750 mW	60 mW/cm^2^	NA
6	Abduljabbar 2017 [64]	Diode laser (HELBO TheraLite Laser, HELBO 3D Pocket Probe; Photodynamic Systems GmbH, Senden, Germany)	660 nm	Phenothiazine chloride/120 s	Applied submucosally from the bottom to the top of the peri-implant pockets	3% Hydrogen peroxide	NO	10 s per site	NA	NA	NA	100 mW	NA	NA
7	Al Amri et al., 2016 [67]	Diode laser (HELBO TheraLite laser HELBO 3D Pocket Probe; Photodynamic Systems GmbH, Senden, Germany)	660 nm	Phenothiazine chloride/120 s	Submucosally, from the bottom to the top of the peri-implant pockets	3% hydrogen peroxide	NO	10 s per site	Inside the peri-implant pocket	NA	NA	100 mW	NA	NA
8	Javed et al., 2016 [68]	Diode laser (HELBO TheraLite laser HELBO 3D Pocket Probe; Photodynamic Systems GmbH, Senden, Germany)	660 nm	Phenothiazine chloride/120 s	Submucosally, from the bottom to the top of the peri-implant pockets	3% hydrogen peroxide	NO	10 s per site	Inside the peri-implant pocket	NA	NA	100 mW	NA	NA
9	Romeo et al., 2016 [69]	Diode laser (HELBO TheraLite, Bredent medical HELBO 3D Pocket Probe; Photodynamic Systems GmbH, Senden, Germany)	670 nm	Phenothiazine chloride/1 min	Applied inside the peri-implant pocket, starting from the bottom and moving in apical coronal direction	NA	NO	1 min	NA	NA	25.54 J/cm^2^	40 mW	75 mW/cm^2^	A spot size of 0.06 cm in diameter
10	Esposito et al., 2013 [70]	Diode Laser (FotoSan CMS Dental, Copenhagen, Denmark)	630 nm	Toluidine blue (0.1 mg/mL)	NA	NA	After 4 months, only if required	80 s in total (20 s at each site around the implant)	A 23 mm long perio tip instrument was inserted at four points around the implant	NA	NA	NA	NA	NA
11	Bombeccari et al., 2013 [36]	Diode laser (Doctor Smile Laser D5-Lambda Scientifica SPA, Vicenza, Italy)	810 nm	Toluidine Blue (100 µg/mL)/1 min	Inside peri-implant pocket	Saline solution	NO	100 s in total (the stained area was irradiated for 20 s for 5 times; in between PDTs, there was 30 s pause)	Along the surfaces of the peri-implant defect	NA	NA	1000 mW	NA	300 μm wave guide fiber

Legend: PDT = photodynamic therapy; NA = not assessed.

**Table 3:** Main characteristics of the selected studies: outcomes, follow-up, methods of evaluation, and conclusions.

	First Author, Publication Year	Probing Depth (mm ± SD)					Marginal Bone Level (mm ± SD)				Bleeding on Probing (±SD)						Plaque Scores (±SD)				Conclusions
1	Alharthi et al., 2022 [71]	Baseline G-I: 5.6 (±1.4) G-II: 5.7 (±0.8) G-III: 5.6 (±0.5) G-IV: 5.7 (±0.6)		9 Months G-I: 4.1 (±0.3) G-II: 0.7 (±0.06) G-III: 0.5 (±0.04) G-IV: 0.4 (±0.004)			Baseline Mesial G-I: 4.5 (±0.3) G-II:4.7 (±0.2) G-III: 4.5 (±0.4) G-IV: 4.7 (±0.3)	Baseline Distal G-I: 4.5(±0.3) G-II:4.7 (±0.2) G-III: 4.5 (±0.4) G-IV: 4.7 (±0.3)	9 Months Mesial G-I: 4.7 (±0.2) G-II: 4.7 (±0.3) G-III: 4.2 (±0.2) G-IV: 4.5 (±0.3)	9 Months Distal G-I: 4.5 (±0.08) G-II: 4.5 (±0.07) G-III: 4.6 (±0.06) G-IV: 4.5 (±0.08)	Baseline G-I: 3.5 (±0.2) G-II: 3.4 (±0.3) G-III: 3.5 (±0.08) G-IV: 3.3 (±0.02)			9 Months G-I: 3.1 (±0.2) G-II: 0.2 (±0.05) G-III: 0.2 (±0.007) G-IV: 0.2 (±0.004)			Baseline G-I: 3.3 (±0.2) G-II: 3.2 (±0.3) G-III: 3.2 (±0.2) G-IV: 3.05 (±0.07)		9 Months G-I: 2.6 (±0.2) G-II: 0.5 (±0.02) G-III: 0.3 (±0.004) G-IV: 0.3 (±0.002)		The use of apdt as an adjunct to MD reduces the severity of peri-implant mucositis but does not contribute towards bone regeneration In peri-implant osseous defects.
2	Labban et al., 2021 [63]	≥6 mm of PD					Peri-implant crestal bone levels (PCBL) change evaluated via digital periapical radiographs														Multiple application of indocyanine-green mediated photodynamic therapy resulted in improved clinical and microbial parameters among type 2 DM subjects in the treatment of peri-implantitis.
		Baseline TG: 6.47 (±0.52) CG: 6.62 (±0.35)		3 Months TG:4.93 (±0.88) CG: 5.17 (±0.79)		6 Months TG: 3.81 (±0.54) CG: 4.93 (±0.87)		3 Months TG: 2.06 (±1.13) CG: 2.19 (±1.42)		6 Months TG: 1.31 (±0.95) CG: 1.92 (±1.13)	Baseline TG:59.12% (±9.37) CG:63.65% (±11.34)			3 Months TG:21.34% (±6.78) CG:42.62% (±12.15)	6 Months TG: 19.19% (±5.98) CG: 31.58% (±15.89)		Baseline TG: 53.98% (±15.18) CG: 57.9% (±9.77)		3 Months TG:17.34% (±4.83) CG:20.45% (±6.31)	6 Months TG: 15.19% (±3.76) CG:17.06% (±4.01)	
3	Ahmed et al., 2020 [65]	≥6 mm of PD on at least one implant site					≥3 mm alveolar bone loss apical to the coronal region of the intraosteal portion of dental implant														The treatment of peri-implantitis using apdt among T2DM patients improved the clinical, radiographic, and immunological peri-implant parameters.
		Baseline G1: 6.8 (±1.4) G2: 6.6 (±1.2) G3: 6.9 (±1.8)		3 Months G1: 4.3 (±0.9) G2: 4.8 (±1.0) G3: 5.4 (±1.7)		6 Months G1: 4.0 (±0.9) G2: 3.9 (±1.0) G3: 4.7 (±1.1)	Baseline G1: 1.9 (±0.4) G2: 2.0(±0.6) G3: 1.8 (±0.5)	3 Months G1: 1.6 (±0.2) G2: 1.7 (±0.3) G3: 1.7 (±0.4)		6 Months G1: 1.0 (±0.6) G2: 0.8 (±0.2) G3: 1.2 (±0.7)	Baseline G1: 52.6% (±25.6) G2:55.8%(±23.5) G3:46.6% (±24.3)			3 Months G1:21.3% (±12.6) G2:25.5% (±13.7) G3:27.7% (±12.6)	6 Months G1:14.5% (±6.7) G2:15.6% (±8.6) G3:19.8% (±9.4)		Baseline G1: 43.3% (±8.6) G2: 45.9% (±9.9) G3: 44.2% (±10.6)		3 Months G1: 17.8% (±4.6) G2: 19.4% (±5.6) G3: 23.3% (±8.4)	6 Months G1: 13.7% (±6.8) G2: 9.4% (±3.4) G3: 14.2% (±6.5)	
4	Alqahtani et al., 2019 [72]	PD ≥ 4 mm					CBL was measured on digital bitewing radiographs. CBL (mesial and/or distal) of ≥3 mm														In the short term, MD with adjunct apdt is effective for the treatment of peri-implantitis. Routine oral hygiene maintenance plays a role in the overall success of MD with or without apdt in patients with peri-implantitis.
		Baseline TG(CS): 5.2 (±0.4) TG (WS): 4.8 (±0.2) TG (NS): 4.5 (±0.2) CG (CS): 5.2 (±0.4) CG (WS): 4.8 (±0.2) CG (NS): 4.5 (±0.2)		3 Months TG (CS): 2.5 (±0.2) TG (WS): 2.6 (±0.3) TG (NS): 2.2 (±0.4) CG (CS): 4.6 (±0.7) CG (WS): 4.1 (±0.5) CG (NS): 3.9 (±0.4)		6 Months TG (CS): 4.6 (±0.2) TG (WS): 4.2 (±0.3) TG (NS): 2.4 (±0.5) CG (CS): 4.4 (±0.3) CG (WS): 4.0 (±0.6) CG (NS): 2.6 (±0.4)	Baseline TG (CS): 5.2 (±0.3) TG (WS): 4.6(±0.3) TG (NS): 4.3 (±0.2) CG (CS): 5.2 (±0.3) CG (WS): 4.6 (±0.3) CG (NS): 4.3 (±0.2)	3 Months TG (CS): 5 (±0.1) TG (WS): 4.6 (±0.2) TG (NS): 3.7 (±0.3) CG (CS): 5 (±0.2) CG (WS): 4.6 (±0.2) CG (NS): 4.3 (±0.3)		6 Months TG (CS): 5 (±0.2) TG (WS): 4.6 (±0.2) TG (NS): 3.3 (±0.4) CG (CS): 5.1 (±0.3) CG (WS): 4.7 (±0.8) CG (NS): 4.1 (±0.2)	Baseline TG (CS): 12.7 (±2.6)% TG (WS): 14.1 (±1.8)% TG (NS): 44.1 (±6.3)% CG (CS): 12.7 (±2.6)% CG (WS): 14.1 (±1.8)% CG (NS): 44.1 (±6.3)%			3 Months TG(CS): 8.1 (±1.2)% TG(WS): 9.3 (±0.8)% TG (NS): 6.1 (±1.2)% CG (CS): 11.2 (±1.7)% CG (WS): 12.5 (±1.6)% CG (NS): 20.9 (±4.3)%	6 Months TG (CS): 12.6 (±3.8)% TG (WS): 13.3 (±6.1)% TG (NS): 8.2 (±1.5)% CG (CS): 13.5 (±5.6)% CG (WS): 12.7 (±5.5)% CG (NS): 20.8 (±4.1)%		Baseline TG (CS): 54.6 (±12.2)% TG (WS): 52.3 (±10.4)% TG (NS): 39.6 (±6.7)% CG (CS): 54.6 (±12.2)% CG (WS): 52.3 (±10.4)% CG (NS): 39.6 (±6.7)%		3 Months TG (CS): 31.3 ± 5.5% TG (WS): 30.5 ± 4.2% TG (NS): 12.4 ± 2.8% CG (CS): 42.5 (±7.9)% CG (WS): 42.1 (±6.4) % CG (NS): 26.5 (±5.7) %	6 Months TG (CS): 46.5 ± 7.3% TG (WS): 44.2 ± 4.8% TG (NS): 14.1 ± 3.1% CG (CS): 43.7 (±8.2) % CG (WS): 40.6 (±9.3) % CG (NS): 23.4 (±3.5)%	
5	Wang et al., 2019 [66]	PD ≥ 6 mm, evaluated in 6 sites around the implants					NA				Sulcus Bleeding Index around the implant						Plaque index				aPDT combined with mechanical debridement significantly improves PD, CAL, PLI, and SBI, compared with baseline and the control group, in participants with peri-implantitis.
		Baseline TG: 4.93 (±1.07) CG: 5.07 (±0.72)		1 Month TG: 4.23 (±0.94) CG: 3.55 (±0.47)	3 Months TG: 3.37 (±0.37) CG: 3.89 (±0.22)	6 Months TG: 3.06 (±0.29) CG: 4.62 (±0.45)					Baseline TC:1.5% grade 3 + 98.5% grade 4 CG: 100% grade 4		1 Month TC: 1.5% grade 1 + 30.8% grade 2 + 37% grade 3 + 10.8% grade 4; CG: 7.6% grade 1; 87.9% grade 2 + 20.02% grade 3	3 Months TC: 58.5% grade 1 + 41.5 grade 2; CG: 45.5% grade 3 + 54.5 grade 3	6 Months TC:1.5% grade 0 + 93.8% grade 1 + 1.5 grade 2 + 3.1 grade 3%; CG: 12.1% grade 2 + 81.8% grade 3 + 6.1% grade 4%		Baseline TC: 3.1% grade 1 + 93.8% grade 2 + 3.1% grade 3 CG: 100% grade 2	1 Month TC: 100% grade 1 CG: 100% grade 1	3 Months TC: 20% grade 0 + 80% grade 1; CG: 100% grade 1	6 Months TC: 27.7% grade 0 + 70.8% grade 1 + 1.5 grade 2 CG: 27.3% grade 1 + 71.2% grade 2 + 1.5% grade 3	
6	Abduljabbar 2017 [64]	PD ≥ 4 mm, 6 sites per implant, and presented as mean percentages per individual					NA				Six sites per implant and presented as mean percentages per individual										In the short-term, MD with adjunct aPDT is more effective in the treatment of peri-implant inflammation compared to MD alone in prediabetic patients.
		Baseline TG: 26.2 (±3.7)% CG: 29.5 (±2.4)%		3 Months TC: 5.1 (±0.8)% CG: 15.5 (±1.4)%		6 Months TC: 8.8 (±0.3)% CG: 10.7 (±0.7)%)					Baseline TC: 30.3% (±4.2) CG: 35.7% (±9.1)			3 Months TC: 8.2% (±4.6) CG: 18.1% (±2.4)	6 Months TC: 10.8% (±0.6) CG: 15.5% (±1.3)		Baseline G1: 43.3% (±8.6) G2: 45.9% (±9.9) G3: 44.2% (±10.6)		3 Months G1: 17.8% (±4.6) G2: 19.4% (±5.6) G3: 23.3% (±8.4)	6 Months G1: 13.7% (±6.8) G2: 9.4% (±3.4) G3: 14.2% (±6.5)	
7	Al Amri et al., 2016 [67]	PD ≥ 4 mm, 6 sites per implant, and presented as mean percentages per individual					Vertical difference between the original peri-implant bone level at baseline and that at follow-up				In at least 30% sites, evaluated in 6 sites around the implant						NA				MD with adjunct apdt is more effective in the treatment of peri-implant inflammation compared with MD alone in patients with T2DM
		Baseline TG: 16.2% (±3.7) CG: 19.5% (±2.4)		6 Months TC: 3.1% (±0.8) CG: 8.5% (±1.4)		12 Months TC: 0.4% (±0.1) CG: 4.3% (±0.7)	Baseline TC: 1.4 (±0.2) CG: 1.3 (±0.6)	6 Months TC: 1.3 (±0.1) CG: 1.4 (±0.1)		12 Months TC: 1.3 (±0.2) CG: 1.3 (±0.1)	Baseline TG: 36.3% (±14.2) CG: 31.7% (±9.4)			6 Months TC: 9.2% (±1.6) CG: 15.1% (±3.4)	12 Months TC: 2.4% (±0.6) CG: 10.5% (±1.3)						
8	Javed et al., 2016 [68]	≥4 mm-Evaluated in 6 sites around the implants					Crestal bone loss (CBL) was defined as the linear distance from the implant–abutment junction to the most coronal part of the alveolar crest. CBL was recorded in millimeters using a software program. No statistically significant difference in CBL among smokers and non-smokers in the test and control groups at all time intervals				In at least 30% sites, 6 sites around the implants						NA				MD with adjunct apdt is more effective in reducing peri-implant probing depth than MD alone in smokers and non-smokers. However, in the long term, outcomes of MD either with or without apdt are comparable among smokers and non-smokers.
		In smoker patients, peri-implant PD was statistically significantly higher at baseline compared with 6- and 12-month follow-up among smokers and non-smokers; significantly higher in the control group compared with the test group (*p* < 0.05). No statistically significant difference in PD among smokers in the test and control groups at 12-month follow-up.									In non-smoker patients at 6-month follow-up, BOP was statistically significantly higher among individuals in the control group compared with the test group. In smokers, no statistically significant difference in BOP between the test and control groups at 6- and 12-month follow-up. In non-smokers, patients’ BOP was significantly higher among individuals in the test and control groups at baseline compared to 6–12 months. At 12-month follow-up, there was no statistically significant difference in BOP.										
9	Romeo et al., 2016 [69]	≥5 mm Evaluated using a plastic probe in 6 sites around the implants					NA				Evaluated in 6 sites around the implants										aPDT with diode laser and phenothiazine chloride represents a reliable adjunctive treatment to conventional therapy in terms of PPD, BOP, and Pl, with an average pocket depth value of 2 mm, when compared with control group (3 mm).
		Baseline TG: 5 CG: 5	6 Weeks TG: 3 CG: 3	3 Months (12 weeks) TC: 2 CG: 2		6 Months (24 weeks) TC: 2 CG: 3					Baseline TG: 100% CG: 100%		6 Weeks TG: 20% CG: 35%	3 Months (12 weeks) TC: 10% CG: 20%	6 Months (24 weeks) TC: 0% CG: 10%		Baseline TC: 60% CG: 62%	1 Month TC: 11% CG: 12%	3 Months (12 weeks) TC: 17% CG: 21%	6 Months (24 weeks) TC: 17% CG: 25%	
10	Esposito et al., 2013 [70]	Evaluated in 4 sites around the implants					≥3 mm of MBL loss; Sites with MBL loss ≤ 5 mm were treated non-surgically, and those with bone loss > 5 mm were treated surgically				Evaluated in 4 sites around the implants										The use of adjunctive LAD therapy with mechanical cleaning of implants affected by peri-implantitis did not improve any clinical outcomes when compared to mechanical cleaning alone up to 1 year after treatment
		Baseline TG: 6.23 (±1.62) CG: 6.45 (±2.15)		4 Months TG: 5.08 (±1.63) CG: 5.25 (±1.63)		1 Year TG: 5.14 (±1.83) CG:5.5.0 (±1.94)	Baseline TG: 4.50 (±1.75) CG: 4.90 (±2.07)	12 MONTHS TG: 4.50 (±1.67) CG: 5.03 (±2.51)			Baseline TG: 2.95% (±1.32) CG: 2.68% (±1.25)	1 Week TG: 1.33% (±1.46) CG: 1.48% (±1.55)		1 Month TG: 1.00% (±1.22) CG: 1.31% (±1.24)	4Months TG: 1.03 (±1.33)%; CG: 1.10 (±1.33)%	1 Year TG: 1.35% (±1.32) CG: 1.28% (±1.11)	Baseline TC: 2.18% (±1.53) CG: 2.15% (±1.64)	1 Month TG: 1.08 (±1.10)% CG: 1.18 (±1.07)%	4 Months TG: 0.97 (±1.14)%; CG: 0.93 (±1.23)%	12 Months TG: 0.89% (±0.94) CG: 0.93% (±0.94)	
11	Bombbeccari et al., 2013 [36]	≥5 mm Evaluated in 4 sites around the implants					All the patients had to have radiographic signs of progressive bone loss (bone loss > 3 threads around the dental implant since at least 12 months)				Evaluated in 4 sites around the implants						NA				The present investigation failed to demonstrate that the treatment of peri-implant lesions with PDT plus TBO by the diode laser leads to a substantial decontamination of anaerobic bacteria on rough titanium implant surfaces, as compared with the conventional surgical aPDT, which resulted in a significant reduction in the bleeding scores and exudates with respect to the conventional surgical approach.
		Baseline TG: 5.9 (±0.76) CG: 5.8 (±0.78)		3 Months TG: 5.2 (±1.03) CG: 5.7 (±0.48)		6 Months TG: 4.9 (±0.47) CG: 5.5 (±0.52)					Baseline TC: 0.70 (±0.48)% CG: 0.80 (±0.44)%			3 Months TC: 0.00 (±0.00)% CG: 0.30 (±0.42)%	6 Months TC: 0.10 (±0.31)% CG: 0.50 (±0.52)%						

Legend: SD = standard deviation; aPDT = antimicrobial photodynamic therapy; PD = probing depth; BOP = bleeding on probing; PI = plaque index; MBL = marginal bone loss; CBL = crestal bone loss; DM = diabetes mellitus; TG = test Group; CG = control group; PS = platform-switched connection; CS = cigarette smokers; WS = waterpipe smokers; NS = never smokers; MD = mechanical debridement; OFD = open flap debridement; LAD = light-activated disinfection; TBO = toluidine blue; NA = not assessed.

## Supplementary Materials

The following supporting information can be downloaded at: https://www.mdpi.com/article/10.3390/dj13120567/s1. Table S1 PRISMA CHECKLIST; Table S2 Excluded articles.

## References

[1] Smeets R., Henningsen A., Jung O., Heiland M., Hammächer C., Stein J.M. (2014). Definition, etiology, prevention and treatment of peri-implantitis—A review. Head Face Med..

[2] Degidi M., Artese L., Piattelli A., Scarano A., Shibli J.A., Piccirilli M., Perrotti V., Iezzi G. (2012). Histological and immunohistochemical evaluation of the peri-implant soft tissues around machined and acid-etched titanium healing abutments: A prospective randomised study. Clin. Oral Investig..

[3] Tonetti M.S., Chapple I.L.C.C., Jepsen S., Sanz M. (2015). Primary and secondary prevention of periodontal and peri-implant diseases: Introduction to, and objectives of the 11th European Workshop on Periodontology consensus conference. J. Clin. Periodontol..

[4] Renvert S., Roos-Jansåker A.-M., Claffey N. (2008). Non-surgical treatment of peri-implant mucositis and peri-implantitis: A literature review. J. Clin. Periodontol..

[5] Rosen P., Clem D., Cochran D., Froum S., McAllister B., Renvert S., Wang H.L. (2013). Peri-implant mucositis and peri-implantitis: A current understanding of their diagnoses and clinical implications. J. Periodontol..

[6] Mombelli A., van Oosten M.A., Schurch E.J., Land N.P. (1987). The microbiota associated with successful or failing osseointegrated titanium implants. Oral Microbiol. Immunol..

[7] Leonhardt A., Bergström C., Lekholm U. (2003). Microbiologic diagnostics at titanium implants. Clin. Implant Dent. Relat. Res..

[8] Fürst M.M., Salvi G.E., Lang N.P., Persson G.R. (2007). Bacterial colonization immediately after installation on oral titanium implants. Clin. Oral Implants Res..

[9] Persson G.R., Samuelsson E., Lindahl C., Renvert S. (2010). Mechanical non-surgical treatment of peri-implantitis: A single-blinded randomized longitudinal clinical study. II. Microbiological results. J. Clin. Periodontol..

[10] Salvi G.E., Fürst M.M., Lang N.P., Persson G.R. (2008). One-year bacterial colonization patterns of Staphylococcus aureus and other bacteria at implants and adjacent teeth. Clin. Oral Implants Res..

[11] Dabdoub S.M., Tsigarida A.A., Kumar P.S. (2013). Patient-specific analysis of periodontal and peri-implant microbiomes. J. Dent. Res..

[12] Lindhe J., Berglundh T., Ericsson I., Liljenberg B., Marinello C. (1992). Experimental breakdown of peri-implant and periodontal tissues. A study in the beagle dog. Clin. Oral Implants Res..

[13] Carcuac O., Abrahamsson I., Albouy J.-P., Linder E., Larsson L., Berglundh T. (2013). Experimental periodontitis and peri-implantitis in dogs. Clin. Oral Implants Res..

[14] Aoki A., Mizutani K., Schwarz F., Sculean A., Yukna R.A., Takasaki A.A., Romanos G.E., Taniguchi Y., Sasaki K.M., Zeredo J.L. (2015). Periodontal and peri-implant wound healing following laser therapy. Periodontol 2000.

[15] Romanos G.E., Javed F., Delgado-Ruiz R.A., Calvo-Guirado J.L. (2015). Peri-implant diseases: A review of treatment interventions. Dent. Clin. N. Am..

[16] Schwarz F., Sahm N., Schwarz K., Becker J. (2010). Impact of defect configuration on the clinical outcome following surgical regenerative therapy of peri-implantitis. J. Clin. Periodontol..

[17] Kotsovilis S., Karoussis I.K., Trianti M., Fourmousis I. (2008). Therapy of peri-implantitis: A systematic review. J. Clin. Periodontol..

[18] Heitz-Mayfield L.J.A., Mombelli A. (2014). The therapy of peri-implantitis: A systematic review. Int. J. Oral. Maxillofac. Implants.

[19] Kolonidis S.G., Renvert S., Hämmerle C.H.F., Lang N.P., Harris D., Claffey N. (2003). Osseointegration on implant surfaces previously contaminated with plaque. An experimental study in the dog. Clin. Oral Implants Res..

[20] Schou S., Berglundh T., Lang N.P. (2004). Surgical treatment of peri-implantitis. Int. J. Oral. Maxillofac. Implants.

[21] Merli M., Bernardelli F., Giulianelli E., Carinci F., Mariotti G., Merli M., Pini-Prato G., Nieri M. (2020). Short-term comparison of two non-surgical treatment modalities of peri-implantitis: Clinical and microbiological outcomes in a two-factorial randomized controlled trial. J. Clin. Periodontol..

[22] Pfitzner A., Sigusch B.W., Albrecht V., Glockmann E. (2004). Killing of periodontopathogenic bacteria by photodynamic therapy. J. Periodontol..

[23] Liñares A., Sanz-Sánchez I., Dopico J., Molina A., Blanco J., Montero E. (2023). Efficacy of adjunctive measures in the non-surgical treatment of peri-implantitis: A systematic review. J. Clin. Periodontol..

[24] Chen J., Keltner L., Christophersen J., Zheng F., Krouse M., Singhal A., Wang S.S. (2002). New technology for deep light distribution in tissue for phototherapy. Cancer J..

[25] Josefsen L.B., Boyle R.W. (2008). Photodynamic therapy: Novel third-generation photosensitizers one step closer?. Br. J. Pharmacol..

[26] Siddiqui S.H., Awan K.H., Javed F. (2013). Bactericidal efficacy of photodynamic therapy against Enterococcus faecalis in infected root canals: A systematic literature review. Photodiagnosis Photodyn. Ther..

[27] Mang T.S., Tayal D.P., Baier R. (2012). Photodynamic therapy as an alternative treatment for disinfection of bacteria in oral biofilms. Lasers Surg. Med..

[28] Prates R.A., Yamada A.M., Suzuki L.C., Hashimoto M.C., Cai S., Gouw-Soares S., Gomes L., Ribeiro M.S. (2007). Bactericidal effect of malachite green and red laser on Actinobacillus actinomycetemcomitans. J. Photochem. Photobiol. B.

[29] Nastri L., Donnarumma G., Porzio C., De Gregorio V., Tufano M.A., Caruso F., Mazza C., Serpico R. (2010). Effects of toluidine blue-mediated photodynamic therapy on periopathogens and periodontal biofilm: In vitro evaluation. Int. J. Immunopathol. Pharmacol..

[30] Annunziata M., Donnarumma G., Guida A., Nastri L., Persico G., Fusco A., Sanz-Sánchez I., Guida L. (2023). Clinical and microbiological efficacy of indocyanine green-based antimicrobial photodynamic therapy as an adjunct to non-surgical treatment of periodontitis: A randomized controlled clinical trial. Clin. Oral. Investig..

[31] Andersen R., Loebel N., Hammond D., Wilson M. (2007). Treatment of periodontal disease by photodisinfection compared to scaling and root planing. J. Clin. Dent..

[32] de Oliveira R.R., Schwartz-Filho H.O., Novaes A.B.J., Taba M.J. (2007). Antimicrobial photodynamic therapy in the non-surgical treatment of aggressive periodontitis: A preliminary randomized controlled clinical study. J. Periodontol..

[33] Salvi G.E., Stähli A., Schmidt J.C., Ramseier C.A., Sculean A., Walter C. (2020). Adjunctive laser or antimicrobial photodynamic therapy to non-surgical mechanical instrumentation in patients with untreated periodontitis: A systematic review and meta-analysis. J. Clin. Periodontol..

[34] Sayar F., Chiniforush N., Bahador A., Etemadi A., Akhondi N., Azimi C. (2019). Efficacy of antimicrobial photodynamic therapy for elimination of Aggregatibacter actinomycetemcomitans biofilm on Laser-Lok titanium discs. Photodiagnosis Photodyn. Ther..

[35] Dörtbudak O., Haas R., Bernhart T., Mailath-Pokorny G. (2001). Lethal photosensitization for decontamination of implant surfaces in the treatment of peri-implantitis. Clin. Oral Implants Res..

[36] Bombeccari G.P., Guzzi G., Gualini F., Gualini S., Santoro F., Spadari F. (2013). Photodynamic therapy to treat periimplantitis. Implant. Dent..

[37] Schär D., Ramseier C.A., Eick S., Arweiler N.B., Sculean A., Salvi G.E. (2013). Anti-infective therapy of peri-implantitis with adjunctive local drug delivery or photodynamic therapy: Six-month outcomes of a prospective randomized clinical trial. Clin. Oral Implants Res..

[38] Bassetti M., Schär D., Wicki B., Eick S., Ramseier C.A., Arweiler N.B., Sculean A., Salvi G.E. (2014). Anti-infective therapy of peri-implantitis with adjunctive local drug delivery or photodynamic therapy: 12-month outcomes of a randomized controlled clinical trial. Clin. Oral Implants Res..

[39] Page M.J., McKenzie J.E., Bossuyt P.M., Boutron I., Hoffmann T.C., Mulrow C.D., Shamseer L., Tetzlaff J.M., Akl E.A., Brennan S.E. (2021). The PRISMA 2020 statement: An updated guideline for reporting systematic reviews. J. Clin. Epidemiol..

[40] Sterne J.A., Savović J., Page M.J., Elbers R.G., Blencowe N.S., Boutron I., Cates C.J., Cheng H.Y., Corbett M.S., Eldridge S.M. (2019). RoB 2: A revised tool for assessing risk of bias in randomised trials. BMJ.

[41] Higgins J.P.T., Thompson S.G., Deeks J.J., Altman D.G. (2003). Measuring inconsistency in meta-analyses. BMJ.

[42] Mongardini C., Pilloni A., Farina R., Di Tanna G., Zeza B. (2017). Adjunctive efficacy of probiotics in the treatment of experimental peri-implant mucositis with mechanical and photodynamic therapy: A randomized, cross-over clinical trial. J. Clin. Periodontol..

[43] Schwarz F., Bieling K., Nuesry E., Sculean A., Becker J. (2006). Clinical and histological healing pattern of peri-implantitis lesions following non-surgical treatment with an Er:YAG laser. Lasers Surg. Med..

[44] Almohareb T., Alhamoudi N., Al Deeb M., Bin-Shuwaish M.S., Mokeem S.A., Shafqat S.S., Vohra F., Abduljabbar T. (2020). Clinical efficacy of photodynamic therapy as an adjunct to mechanical debridement in the treatment of per-implantitis with abscess. Photodiagnosis Photodyn. Ther..

[45] Al-Askar M.H., Abdullatif F.A., Alshihri A.A., Ahmed A., Divakar D.D., Almoharib H., Alzoman H. (2022). Comparison of photobiomodulation and photodynamic therapy as adjuncts to mechanical debridement for the treatment of peri-implantitis. Technol. Health Care Off. J. Eur. Soc. Eng. Med..

[46] Al Deeb M., Alresayes S., Mokeem S.A., Alhenaki A.M., AlHelal A., Shafqat S.S., Vohra F., Abduljabbar T. (2020). Clinical and immunological peri-implant parameters among cigarette and electronic smoking patients treated with photochemotherapy: A randomized controlled clinical trial. Photodiagnosis Photodyn. Ther..

[47] Shetty B., Ali D., Ahmed S., Ibraheem W.I., Preethanath R.S., Vellappally S., Divakar D.D. (2022). Role of antimicrobial photodynamic therapy in reducing subgingival oral yeasts colonization in patients with peri-implant mucositis. Photodiagnosis Photodyn. Ther..

[48] Deeb MAl Alsahhaf A., Mubaraki S.A., Alhamoudi N., Al-Aali K.A., Abduljabbar T. (2020). Clinical and microbiological outcomes of photodynamic and systemic antimicrobial therapy in smokers with peri-implant inflammation. Photodiagnosis Photodyn. Ther..

[49] Al Rifaiy M.Q., Qutub O.A., Alasqah M.N., Al-Sowygh Z.H., Mokeem S.A., Alrahlah A. (2018). Effectiveness of adjunctive antimicrobial photodynamic therapy in reducing peri-implant inflammatory response in individuals vaping electronic cigarettes: A randomized controlled clinical trial. Photodiagnosis Photodyn. Ther..

[50] Al-Sowygh Z.H. (2017). Efficacy of periimplant mechanical curettage with and without adjunct antimicrobial photodynamic therapy in smokeless-tobacco product users. Photodiagnosis Photodyn. Ther..

[51] Zeza B., Farina R., Pilloni A., Mongardini C. (2018). Clinical outcomes of experimental gingivitis and peri-implant mucositis treatment with professionally administered plaque removal and photodynamic therapy. Int. J. Dent. Hyg..

[52] Ohba S., Sato M., Noda S., Yamamoto H., Egahira K., Asahina I. (2020). Assessment of safety and efficacy of antimicrobial photodynamic therapy for peri-implant disease. Photodiagnosis Photodyn. Ther..

[53] Karimi M.R., Hasani A., Khosroshahian S. (2016). Efficacy of antimicrobial photodynamic therapy as an adjunctive to mechanical debridement in the treatment of peri-implant diseases: A randomized controlled clinical trial. J. Lasers Med. Sci..

[54] De Angelis N., Felice P., Grusovin M.G., Camurati A., Esposito M. (2012). The effectiveness of adjunctive light-activated disinfection (LAD) in the treatment of periimplantitis: 4-month results from a multicentre pragmatic randomised controlled trial. Eur. J. Oral Implantol..

[55] ALHarthi S.S., Divakar D.D., Alwahibi A., BinShabaib M.S. (2022). Effect of mechanical instrumentation with adjunct photodynamic therapy on salivary TNFα levels and clinical periodontal and peri-implant status in patients with depression: A randomized controlled trial. Photodiagnosis Photodyn. Ther..

[56] Rakašević D., Lazić Z., Rakonjac B., Soldatović I., Janković S., Magić M., Aleksić Z. (2016). Efficiency of photodynamic therapy in the treatment of peri-implantitis—A three-month randomized controlled clinical trial. Srp. Arh. Celok. Lek..

[57] Albaker A.M., ArRejaie A.S., Alrabiah M., Al-Aali K.A., Mokeem S., Alasqah M.N., Vohra F., Abduljabbar T. (2018). Effect of antimicrobial photodynamic therapy in open flap debridement in the treatment of peri-implantitis: A randomized controlled trial. Photodiagnosis Photodyn. Ther..

[58] Abduljabbar T. (2017). Effect of mechanical debridement with adjunct antimicrobial photodynamic therapy in the treatment of peri-implant diseases in type-2 diabetic smokers and non-smokers. Photodiagnosis Photodyn. Ther..

[59] Rakasevic D., Lazic Z., Aleksic Z., Jankovic S., Nikolic N.J., Soldatovic I., Djukic L. (2019). Evaluation of clinical and immunological parameters after applying the adjunctive photodynamic therapy in the surgical treatment of peri-implantitis. A 6- and 12-month randomized controlled clinical trial. Clin. Oral Implants Res..

[60] Lazic Z., Jankovic S.M., Jakoba N.N., Soldatovic I., Roganovic J., Aleksic Z.M. (2018). Clinical and immunological response to photodynamic therapy in the treatment of peri-implantitis. J. Clin. Periodontol..

[61] Leretter M., Cândea A., Topala L. Photodynamic Therapy in Peri-implantitis. Proceedings of the Fifth International Conference on Lasers in Medicine: Biotechnologies Integrated in Daily Medicine.

[62] Bianchine G.M., Fischer R.G., Oliveira L.S. (2018). PR389: Clinical effects of antimicrobial photodynamic therapy as an adjunctive to mechanical debridement in the treatment of peri-implantitis: Preliminary results from a randomized controlled clinical trial. J. Clin. Periodontol..

[63] Labban N., Shibani NAl Al-Kattan R., Alfouzan A.F., Binrayes A., Assery M.K. (2021). Clinical, bacterial, and inflammatory outcomes of indocyanine green-mediated photodynamic therapy for treating periimplantitis among diabetic patients: A randomized controlled clinical trial. Photodiagnosis Photodyn. Ther..

[64] Abduljabbar T. (2017). Effect of mechanical debridement with and without adjunct antimicrobial photodynamic therapy in the treatment of peri-implant diseases in prediabetic patients. Photodiagnosis Photodyn. Ther..

[65] Ahmed P., Bukhari I.A., Albaijan R., Sheikh S.A., Vohra F. (2020). The effectiveness of photodynamic and antibiotic gel therapy as an adjunct to mechanical debridement in the treatment of peri-implantitis among diabetic patients. Photodiagnosis Photodyn. Ther..

[66] Wang H., Li W., Zhang D., Li W., Wang Z. (2019). Adjunctive photodynamic therapy improves the outcomes of peri-implantitis: A randomized controlled trial. Aust. Dent. J..

[67] Al Amri M.D., Kellesarian S.V., Ahmed A., Al-Kheraif A.A., Romanos G.E., Javed F. (2016). Efficacy of periimplant mechanical debridement with and without adjunct antimicrobial photodynamic therapy in patients with type 2 diabetes mellitus. Photodiagnosis Photodyn. Ther..

[68] Javed F., Abduljabbar T., Carranza G., Gholamiazizi E., Mazgaj D.K., Kellesarian S.V., Vohra F. (2016). Efficacy of periimplant mechanical debridement with and without adjunct antimicrobial photodynamic therapy in the treatment of periimplant diseases among cigarette smokers and non-smokers. Photodiagnosis Photodyn. Ther..

[69] Romeo U., Nardi G.M., Libotte F., Sabatini S., Palaia G., Grassi F.R. (2016). The antimicrobial photodynamic therapy in the treatment of peri-implantitis. Int. J. Dent..

[70] Esposito M., Grusovin M.G., De Angelis N., Camurati A., Campailla M., Felice P. (2013). The adjunctive use of light-activated disinfection (LAD) with FotoSan is ineffective in the treatment of peri-implantitis: 1-year results from a multicentre pragmatic randomised controlled trial. Eur. J. Oral Implantol..

[71] ALHarthi S.S., Alamry N.Z., BinShabaib M.S. (2022). Effect of multiple sessions of photodynamic therapy on bone regeneration around dental implants among patients with peri-implantitis. Photodiagnosis Photodyn. Ther..

[72] Alqahtani F., Alqhtani N., Alkhtani F., Divakar D.D., Al-Kheraif A.A., Javed F. (2019). Efficacy of mechanical debridement with and without adjunct antimicrobial photodynamic therapy in the treatment of peri-implantitis among moderate cigarette-smokers and waterpipe-users. Photodiagnosis Photodyn. Ther..

[73] Schwarz F., Derks J., Monje A., Wang H.-L. (2018). Peri-implantitis. J. Clin. Periodontol..

[74] Sanz M., Chapple I.L., on behalf of Working Group 4 of the VIII European Workshop on Periodontology* (2012). Clinical research on peri-implant diseases: Consensus report of Working Group 4. J. Clin. Periodontol..

[75] Stavropoulos A., Bertl K., Winning L., Polyzois I. (2021). What is the influence of implant surface characteristics and/or implant material on the incidence and progression of peri-implantitis? A systematic literature review. Clin. Oral Implants Res..

[76] Mattheos N., Janda M., Acharya A., Pekarski S., Larsson C. (2021). Impact of design elements of the implant supracrestal complex (ISC) on the risk of peri-implant mucositis and peri-implantitis: A critical review. Clin. Oral Implants Res..

[77] Klinge B., Meyle J. (2012). Peri-implant tissue destruction. The Third EAO Consensus Conference 2012. Clin. Oral Implants Res..

[78] Berglundh T., Armitage G., Araujo M.G., Avila-Ortiz G., Blanco J., Camargo P.M., Chen S., Cochran D., Derks J., Figuero E. (2018). Peri-implant diseases and conditions: Consensus report of workgroup 4 of the 2017 World Workshop on the Classification of Periodontal and Peri-Implant Diseases and Conditions. J. Periodontol..

[79] Saneja R., Bhattacharjee B., Bhatnagar A., Kumar P.G.N., Verma A. (2020). Efficacy of different lasers of various wavelengths in treatment of peri-implantitis and peri-implant mucositis: A systematic review and meta-analysis. J. Indian Prosthodont. Soc..

[80] Cosola S., Oldoini G., Giammarinaro E., Covani U., Genovesi A., Marconcini S. (2022). The effectiveness of the information-motivation model and domestic brushing with a hypochlorite-based formula on peri-implant mucositis: A randomized clinical study. Clin. Exp. Dent. Res..

[81] Chala M., Anagnostaki E., Mylona V., Chalas A., Parker S., Lynch E. (2020). Adjunctive Use of Lasers in Peri-Implant Mucositis and Peri-Implantitis Treatment: A Systematic Review. Dent. J..

[82] Chambrone L., Wang H.-L., Romanos G.E. (2018). Antimicrobial photodynamic therapy for the treatment of periodontitis and peri-implantitis: An American Academy of Periodontology best evidence review. J. Periodontol..

[83] Shahmohammadi R., Younespour S., Paknejad M., Chiniforush N., Heidari M. (2022). Efficacy of Adjunctive Antimicrobial Photodynamic Therapy to Mechanical Debridement in the Treatment of Peri-implantitis or Peri-implant Mucositis in Smokers: A Systematic Review and Meta-analysis. Photochem. Photobiol..

