# Supplementary material for: The Effect of Adjunctive Antimicrobial Photodynamic Therapy in the Treatment of Peri-Implant Diseases: Systematic Review and Meta-Analysis
Source: Dent J (Basel). 2025 Dec 1;13(12):567. doi: 10.3390/dj13120567 (PMC12731462; doi:10.3390/dj13120567)
Supplement: Supplementary file 1 [file dentistry-13-00567-s001.zip › Table S2. Excluded Articles.pdf]

Supplementary TABLE S2

	Excluded Items
1.	Abduljabbar T. Effect of mechanical debridement with adjunct antimicrobial photodynamic therapy in the treatment of peri-implant diseases in type-2 diabetic smokers and non-smokers. Photodiagnosis Photodyn Ther. 2017 Mar;17:111-114. doi: 10.1016/j.pdpdt.2016.11.005. Epub 2016 Nov 21. PMID: 27884740.<br>Reason: not related to clinical question
2.	Abohabib AM, Fayed MM, Labib AH. Effects of low-intensity laser therapy on the stability of orthodontic mini-implants: a randomised controlled clinical trial. J Orthod. 2018 Sep;45(3):149-156. doi: 10.1080/14653125.2018.1481710. Epub 2018 Jun 6. PMID: 29874972.<br>Reason: not related to clinical question
3.	Adly MS, Adly AS, Rasheed AM, Adly AS. CAN COMBINING LOW LEVEL LASER THERAPY WITH COMPUTER GUIDED FLAPLESS PIEZOSURGICAL OSTEOTOMY ACHIEVE A PAINLESS IMPLANT SURGERY? FINDINGS OF SPLIT MOUTH RANDOMIZED CONTROLLED TRIAL. J Evid Based Dent Pract. 2022 Sep;22(3):101730. doi: 10.1016/j.jebdp.2022.101730. Epub 2022 Apr 18. PMID: 36162887.<br>Reason: not related to clinical question
4.	Afrasiabi S, Barikani HR, Chiniforush N. Comparison of bacterial disinfection efficacy using blue and red lights on dental implants contaminated with Aggregatibacter actinomycetemcomitans. Photodiagnosis Photodyn Ther. 2022 Dec;40:103178. doi: 10.1016/j.pdpdt.2022.103178. Epub 2022 Oct 29. PMID: 36602065.<br>Reason: in vitro study
5.	Ahamed AS, Prakash PSG, Crena J, Victor DJ, Subramanian S, Appukuttan D. The influence of laser-microgrooved implant and abutment surfaces on mean crestal bone levels and peri-implant soft tissue healing: a 3-year longitudinal randomized controlled clinical trial. Int J Implant Dent. 2021 Oct 4;7(1):102. doi: 10.1186/s40729-021-00382-3. PMID: 34604936; PMCID: PMC8487924.<br>Reason: not related to clinical question
6.	Aimetti M, Mariani GM, Ferrarotti F, Ercoli E, Liu CC, Romano F. Adjunctive efficacy of diode laser in the treatment of peri-implant mucositis with mechanical therapy: A randomized clinical trial. Clin Oral Implants Res. 2019 May;30(5):429-438. doi: 10.1111/clr.13428. Epub 2019 Apr 16. PMID: 30933384.<br>Reason: Studies utilizing low level laser therapy or laser therapy alone
7.	Al Deeb M, Alresayes S, A Mokeem S, Alhenaki AM, AlHelal A, Shafqat SS, Vohra F, Abduljabbar T. Clinical and immunological peri-implant parameters among cigarette and electronic smoking patients treated with photochemotherapy: A randomized controlled clinical trial. Photodiagnosis Photodyn Ther. 2020 Sep;31:101800. doi: 10.1016/j.pdpdt.2020.101800. Epub 2020 Apr 29. PMID: 32360852.<br>Reason: insufficient follow-up
8.	Al Rifaiy MQ, Qutub OA, Alasqah MN, Al-Sowygh ZH, Mokeem SA, Alrahlah A. Effectiveness of adjunctive antimicrobial photodynamic therapy in reducing peri- implant inflammatory response in individuals vaping electronic cigarettes: A randomized controlled clinical trial. Photodiagnosis Photodyn Ther. 2018 Jun;22:132-136. doi: 10.1016/j.pdpdt.2018.03.002. Epub 2018 Mar 15. PMID:29550362.<br>Reason: insufficient follow-up
9.	Alam MK, Alqahtani AA, Zaman MU, Kanwal B, Robaian A, Alqahtani F. Clinical and radiographic outcomes of adjunctive photodynamic therapy for treating peri-implantitis among diabetics and cigarette smokers: a systematic review of randomized controlled trials. Lasers Med Sci. 2023 Jun 19;38(1):142. doi: 10.1007/s10103-023-03807-0. PubMed PMID: 37335369.<br>Reason: Review
10.	Al-Askar MH, Abdullatif FA, Alshihri AA, et al. Comparison of photobiomodulation and

	photodynamic therapy as adjuncts to mechanical debridement for the treatment of peri-implantitis. *Technol Heal care Off J Eur Soc Eng Med*. 2022;30(2):389-398. doi:10.3233/THC-213062<br>Reason: insufficient follow-up0
11.	Albaker AM, ArRejaie AS, Alrabiah M, Al-Aali KA, Mokeem S, Alasqah MN, Vohra F, Abduljabbar T. Effect of antimicrobial photodynamic therapy in open flap debridement in the treatment of peri-implantitis: A randomized controlled trial. Photodiagnosis Photodyn Ther. 2018 Sep;23:71-74. doi: 10.1016/j.pdpdt.2018.05.003. Epub 2018 May 5. PMID: 29738817.<br>Reason: Studies in which aPDT was done after surgery
12.	Albeshri S, AlRowis R. Effect of mechanical debridement with and without adjunct antimicrobial photodynamic therapy for the treatment of peri-implant disease in obese patients: A systematic review and meta-analysis of randomized controlled trials. Photodiagnosis Photodyn Ther. 2025 Apr;52:104510. doi: 10.1016/j.pdpdt.2025.104510. Epub 2025 Feb 1. Review. PubMed PMID: 39894321.<br>Reason: Review
13.	Al-Delayme RMA. Preservation of keratinized gingiva around dental implants using a diode laser when uncovering implants for second stage surgery. Eur Oral Res. 2019 Sep;53(3):106-112. doi: 10.26650/eor.20190022. Epub 2019 Sep 1. PMID: 31579890; PMCID: PMC6761491.<br>Reason: not related to clinical question
14.	ALHarthi SS, Divakar DD, Alwahibi A, BinShabaib MS. Effect of mechanical instrumentation with adjunct photodynamic therapy on salivary TNFα levels and clinical periodontal and peri-implant status in patients with depression: A randomized controlled trial. Photodiagnosis Photodyn Ther. 2022 Dec;40:103042. doi: 10.1016/j.pdpdt.2022.103042. Epub 2022 Jul 29. PMID: 35908682.<br>Reason: insufficient follow-up
15.	Ali D, Alsalman J. Efficacy of mechanical debridement with adjunct antimicrobial photodynamic therapy against peri-implant subgingival oral yeasts colonization: A systematic review and meta-analysis. Photodiagnosis Photodyn Ther. 2024 Dec;50:104399. doi: 10.1016/j.pdpdt.2024.104399. Epub 2024 Nov 10. Review. PubMed PMID: 39532194.<br>Reason: Review
16.	Al-Khayatt AS, Eliyas S. Soft tissue handling during implant placement. Evid Based Dent. 2008;9(3):77. doi: 10.1038/sj.ebd.6400596. PMID: 18927566.<br>Reason: not related to clinical question
17.	Almohareb T, Alhamoudi N, Al Deeb M, Bin-Shuwaish MS, Mokeem SA, Saad Shafqat S, Vohra F, Abduljabbar T. Clinical efficacy of photodynamic therapy as an adjunct to mechanical debridement in the treatment of per-implantitis with abscess. Photodiagnosis Photodyn Ther. 2020 Jun;30:101750. doi: 10.1016/j.pdpdt.2020.101750. Epub 2020 Apr 25. PMID: 32545150.<br>Reason: Studies using additional antibiotic or other adjunctive therapy in the test or control group
18.	AlMoharib HS, Steffensen B, Zoukhri D, Finkelman M, Gyurko R. Efficacy of an Er:YAG laser in the decontamination of dental implant surfaces: An in vitro study. J Periodontol. 2021 Nov;92(11):1613-1621. doi: 10.1002/JPER.20-0765. Epub 2021 Apr 6. PMID: 33687796.<br>Reason: Studies utilizing low level laser therapy or laser therapy alone
19.	AlMubarak AM. Comparison of local minocycline hydrochloride delivery and antimicrobial photodynamic therapy as adjuncts to mechanical debridement for the treatment of peri-implant mucositis: A randomized controlled trial. Photodiagnosis Photodyn Ther. 2025 Feb;51:104461. doi: 10.1016/j.pdpdt.2024.104461. Epub 2024 Dec 27. PubMed PMID: 39734027.<br>Reason: Insufficient follow-up
20.	Alpaslan Yayli NZ, Talmac AC, Keskin Tunc S, Akbal D, Altindal D, Ertugrul AS. Erbium, chromium-doped: yttrium, scandium, gallium, garnet and diode lasers in the treatment of peri-implantitis: clinical and biochemical outcomes in a randomized-controlled clinical trial. Lasers Med Sci. 2022 Feb;37(1):665-674. doi: 10.1007/s10103-021-03436-5. Epub 2021 Oct 12. PMID:

	34637055.<br>Reason: Studies utilizing low level laser therapy or laser therapy alone
21.	Alqahtani F, Alqhtani N, Celur SL, Divakar DD, Al-Kheraif AA, Alkhtani F. Efficacy of Nonsurgical Mechanical Debridement With and Without Adjunct Low-Level Laser Therapy in the Treatment of Peri-Implantitis: A Randomized Controlled Trial. J Oral Implantol. 2020 Oct 1;46(5):526-531. doi: 10.1563/aaid-joi-D-19-00367. PMID: 32369570.<br>Reason: Studies utilizing low level laser therapy or laser therapy alone
22.	Alresheedi B, Alazmi S. Disinfection of implant abutment connection using antimicrobial photodynamic therapy and 0.2% chlorhexidine gel applications immediately before prosthesis delivery: Clinical and radiographic status at 1-year of follow-up. Photodiagnosis Photodyn Ther. 2022 Jun;38:102790. doi: 10.1016/j.pdpdt.2022.102790. Epub 2022 Mar 1. PMID: 35245672.<br>Reason: not related to clinical question
23.	Alsayed H, Bukhari IA, Alsaif R, Vohra F. Efficacy of indocyanine green and methylene blue mediated-photodynamic therapy on peri-implant outcomes among diabetics with peri-implant mucositis. Photodiagnosis Photodyn Ther. 2023 Jun;42:103344. doi: 10.1016/j.pdpdt.2023.103344. Epub 2023 Feb 24. PubMed PMID: 36841279.<br>Reason: Insufficient follow-up
24.	Al-Sowygh ZH. Efficacy of periimplant mechanical curettage with and without adjunct antimicrobial photodynamic therapy in smokeless-tobacco product users. Photodiagnosis Photodyn Ther. 2017 Jun;18:260-263. doi: 10.1016/j.pdpdt.2017.03.011. Epub 2017 Mar 24. PMID: 28347865.<br>Reason: insufficient follow-up
25.	Arısan V, Karabuda ZC, Arıcı SV, Topçuoğlu N, Külekçi G. A randomized clinical trial of an adjunct diode laser application for the nonsurgical treatment of peri-implantitis. Photomed Laser Surg. 2015 Nov;33(11):547-54. doi: 10.1089/pho.2015.3956. Epub 2015 Sep 18. PMID: 26382562; PMCID: PMC4649765.<br>Reason: Studies utilizing low level laser therapy or laser therapy alone
26.	Arnabat-Domínguez J, España-Tost AJ, Berini-Aytés L, Gay-Escoda C. Erbium:YAG laser application in the second phase of implant surgery: a pilot study in 20 patients. Int J Oral Maxillofac Implants. 2003 Jan-Feb;18(1):104-12. PMID: 12608675.<br>Reason: not related to clinical question
27.	Aseri AA. Clinical and Radiographic Outcomes of Adjunctive Phototherapy Versus Antibiotic Therapy Against Peri-Implant Diseases: A Systematic Review and Meta-Analysis. Photobiomodul Photomed Laser Surg. 2024 Mar;42(3):189-199. doi: 10.1089/photob.2023.0157. Review. PubMed PMID: 38512322.<br>Reason: Review
28.	Bach G, Neckel C, Mall C, Krekeler G. Conventional versus laser-assisted therapy of periimplantitis: a five-year comparative study. Implant Dent. 2000;9(3):247-51. doi: 10.1097/00008505-200009030-00010. PMID: 11307411.<br>Reason: Studies utilizing low level laser therapy or laser therapy alone
29.	Bahrami R, Nikparto N, Gharibpour F, Pourhajibagher M, Bahador A. Antimicrobial photodynamic therapy for managing the peri-implant mucositis and peri-implantitis: A systematic review of randomized clinical trials. Photodiagnosis Photodyn Ther. 2024 Feb;45:103990. doi: 10.1016/j.pdpdt.2024.103990. Epub 2024 Jan 24. Review. PubMed PMID: 38278339.<br>Reason: Review
30.	Bassetti M, Schär D, Wicki B, Eick S, Ramseier CA, Arweiler NB, Sculean A, Salvi GE. Anti-infective therapy of peri-implantitis with adjunctive local drug delivery or photodynamic therapy: 12-month outcomes of a randomized controlled clinical trial. Clin Oral Implants Res. 2014 Mar;25(3):279-287. doi:10.1111/clr.12155. Epub 2013 Apr 8. PMID: 23560645.<br>Reason: Studies using additional antibiotic or other adjunctive therapy in the test or control group

Supplementary TABLE S2

31.	Bianchine G.M., R.G. Fischer, L.S. Oliveira. Clinical effects of antimicrobial photodynamic therapy as an adjunctive to mechanical debridement in the treatment of peri-implantitis: preliminary results from a randomized controlled clinical Trial. 2018 European Federation of Periodontology. E-Poster<br>Reason: poster
32.	Birang E, Talebi Ardekani MR, Rajabzadeh M, Sarmadi G, Birang R, Gutknecht N. Evaluation of Effectiveness of Photodynamic Therapy With Low-level Diode Laser in Nonsurgical Treatment of Peri-implantitis. J Lasers Med Sci. 2017 Summer;8(3):136-142. doi: 10.15171/jlms.2017.25. Epub 2017 Jun 27. PMID: 29123634; PMCID: PMC5662503.<br>Reason: Studies utilizing low level laser therapy or laser therapy alone
33.	Bitencourt FV, Cardoso De David S, Schutz JDS, Otto Kirst Neto A, Visioli F, Fiorini T. Minimizing patient morbidity after free gingival graft harvesting: A triple-blind randomized-controlled clinical trial. Clin Oral Implants Res. 2022 Jun;33(6):622-633. doi: 10.1111/clr.13923. Epub 2022 Apr 6. PMID: 35305280.<br>Reason: not related to clinical question:
34.	Blanco C, Pico A, Dopico J, Gándara P, Blanco J, Liñares A. Adjunctive benefits of systemic metronidazole on non-surgical treatment of peri-implantitis. A randomized placebo-controlled clinical trial. J Clin Periodontol. 2022 Jan;49(1):15-27. doi: 10.1111/jcpe.13564. Epub 2021 Oct 28. PMID: 34713471.<br>Reason: Not related to clinical question
35.	Bozkaya S, Uraz A, Guler B, Kahraman SA, Turhan Bal B. The stability of implants and microbiological effects following photobiomodulation therapy with one-stage placement: A randomized, controlled, single-blinded, and split-mouth clinical study. Clin Implant Dent Relat Res. 2021 Jun;23(3):329-340. doi: 10.1111/cid.12999. Epub 2021 Apr 14. PMID: 33851765.<br>Reason: not related to clinical question
36.	Canullo L, Tallarico M, Penarrocha M, Corrente G, Fiorellini J, Penarrocha D. Plasma of Argon Cleaning Treatment on Implant Abutments in Periodontally Healthy Patients: Six Years Postloading Results of a Randomized Controlled Trial. Int J Periodontics Restorative Dent. 2017 Sep/Oct;37(5):683-690. doi: 10.11607/prd.3079. PMID: 28817132.<br>Reason: not related to clinical question
37.	Chala M, Anagnostaki E, Mylona V, Chalas A, Parker S, Lynch E. Adjunctive Use of Lasers in Peri-Implant Mucositis and Peri-Implantitis Treatment: A Systematic Review. Dent J (Basel). 2020 Jul 3;8(3):68. doi: 10.3390/dj8030068. PMID: 32635258; PMCID: PMC7560070.<br>Reason: Review
38.	Chen JH, Lin YC, Kung JC, Yan DY, Chen IH, Jheng YS, Lai CH, Wu YM, Lee KT. Efficacy of Er:YAG laser for the peri-implantitis treatment and microbiological changes: a randomized controlled trial. Lasers Med Sci. 2022 Dec;37(9):3517-3525. doi: 10.1007/s10103-022-03627-8. Epub 2022 Aug 11. PMID:35951124.<br>Reason: Studies utilizing low level laser therapy or laser therapy alone
39.	Costa ACF, Maia TAC, de Barros Silva PG, Abreu LG, Gondim DV, Santos PCF. Effects of low-level laser therapy on the orthodontic mini-implants stability: a systematic review and meta-analysis. Prog Orthod. 2021 Feb 15;22(1):6. doi: 10.1186/s40510-021-00350-y. PMID: 33586080; PMCID: PMC7882650.<br>Reason: Review
40.	Davoudi A, Ebadian B, Nosouhian S. Role of laser or photodynamic therapy in treatment of denture stomatitis: A systematic review. J Prosthet Dent. 2018 Oct;120(4):498-505. doi: 10.1016/j.prosdent. 2018.01.003. Epub 2018 May 25. PMID: 29807743.<br>Reason: not related to clinical question
41.	De Angelis N, Felice P, Grusovin MG, Camurati A, Esposito M. The effectiveness of adjunctive light-activated disinfection (LAD) in the treatment of peri-implantitis: 4-month results from a multicentre pragmatic randomised controlled trial. Eur J Oral Implantol. 2012 Winter;5(4):321-31. PMID: 23304686.<br>Reason: insufficient follow-up

42.	Deeb MA, Alsahhaf A, Mubaraki SA, Alhamoudi N, Al-Aali KA, Abduljabbar T. Clinical and microbiological outcomes of photodynamic and systemic antimicrobial therapy in smokers with peri-implant inflammation. Photodiagnosis Photodyn Ther. 2020 Mar;29:101587. doi: 10.1016/j.pdpdt.2019.101587. Epub 2019 Nov 2. PMID:31689510.<br>Reason: insufficient follow-up
43.	Deppe H, Mücke T, Wagenpfeil S, Kesting M, Sculean A. Nonsurgical antimicrobial photodynamic therapy in moderate vs severe peri-implant defects: a clinical pilot study. Quintessence Int. 2013;44(8):609-18. doi: 10.3290/j.qi.a29505. PMID: 23534047.<br>Reason: not randomized clinical trial
44.	Dharmarajan L, Prakash PSG, Appukuttan D, Crena J, Subramanian S, Alzahrani KJ, Alsharif KF, Halawani IF, Alnfiai MM, Alamoudi A, Kamil MA, Balaji TM, Patil S. The Effect of Laser Micro Grooved Platform Switched Implants and Abutments on Early Crestal Bone Levels and Peri-Implant Soft Tissues Post 1 Year Loading among Diabetic Patients-A Controlled Clinical Trial. Medicina (Kaunas). 2022 Oct 15;58(10):1456. doi: 10.3390/medicina58101456. PMID: 36295619; PMCID:PMC9609409.<br>Reason: not related to clinical question
45.	Dominique C, Rethoré G, Verner C, Alliot C, Soueidan A, Struillou X. Use of Lasers in the Nonsurgical Treatment of Peri-Implantitis: A Systematic Review of the Literature. J Oral Implantol. 2024 Oct 1;50(5):552-560. doi: 10.1563/aaid-joi-D-23-00028. PubMed PMID: 38867374.<br>Reason: Review
46.	Dommisch H, Hoedke D, Valles C, Vilarrasa J, Jepsen S, Pascual La Rocca A. Efficacy of professionally administered chemical agents as an adjunctive treatment to sub-marginal instrumentation during the therapy of peri-implant mucositis. J Clin Periodontol. 2022 Nov 14. doi: 10.1111/jcpe.13747. Epub ahead of print. PMID: 36375839.<br>Reason: Review
47.	Dörtbudak O, Haas R, Bernhart T, Mailath-Pokorny G. Lethal photosensitization for decontamination of implant surfaces in the treatment of peri-implantitis. Clin Oral Implants Res. 2001 Apr;12(2):104-8. doi: 10.1034/j.1600-0501.2001.012002104.x. PMID: 11251658.<br>Reason: not randomized clinical trial
48.	Elsadek MF. Effectiveness of two photosensitizer-mediated photodynamic therapy for treating moderate peri-implant infections in type-II diabetes mellitus patients: A randomized clinical trial. Photodiagnosis Photodyn Ther. 2023 Sep;43:103643. doi: 10.1016/j.pdpdt.2023.103643. Epub 2023 Jun 1. PubMed PMID: 37270045.<br>Reason: Not related to clinical question
49.	ELsyad MA, Abdraboh AE, Aboelnagga MM, Ghali RM, Lebshtien IT. Effect of Low-Level Laser Irradiation on Stability and Marginal Bone of Narrow Implants. Retaining Overdentures in Moderately Controlled Diabetic Patients. J Oral Implantol. 2019 Oct;45(5):391-397. doi: 10.1563/aaid-joi-D-18-00263. Epub 2019 Aug 7. PMID: 31389750.<br>Reason: not related to clinical question
50.	Erduran NE, Guncu GN, Akman AC, Acar B, Pinar A, Karabulut E, Nohutcu RM. Evaluation of the effect of adjunctive diode laser application on peri-implant crevicular fluid biomarker levels: a randomized controlled trial. Clin Oral Investig. 2024 Jul 31;28(8):459. doi: 10.1007/s00784-024-05855-4. PubMed PMID: 39080143; PubMed Central PMCID: PMC11289239.<br>Reason: Not related to clinical question
51.	Esposito M, Grusovin MG, Coulthard P, Worthington HV. Interventions for replacing missing teeth: treatment of perimplantitis. Cochrane Database Syst Rev. 2006 Jul 19;(3):CD004970. doi: 10.1002/14651858.CD004970.pub2. Update in: Cochrane Database Syst Rev. 2008;(2):CD004970. PMID: 16856071.<br>Reason: Review
52.	Esposito M, Grusovin MG, Coulthard P, Worthington HV. The efficacy of interventions to treat peri-implantitis: a Cochrane systematic review of randomised controlled clinical trials. Eur J

	Oral Implantol. 2008 Summer;9 Suppl 1(2):111-25. PMID: 20467649.<br>Reason: Review
53.	Esposito M, Grusovin MG, Kakisis I, Coulthard P, Worthington HV. Interventions for replacing missing teeth: treatment of perimplantitis. Cochrane Database Syst Rev. 2008 Apr 16;(2):CD004970. doi: 10.1002/14651858.CD004970.pub3. Update in: Cochrane Database Syst Rev. 2010;(6):CD004970. PMID: 18425907.<br>Reason: Review
54.	Esposito M, Grusovin MG, Maghaireh H, Coulthard P, Worthington HV. Interventions for replacing missing teeth: management of soft tissues for dental implants. Cochrane Database Syst Rev. 2007 Jul 18;(3):CD006697. doi: 10.1002/14651858.CD006697. Update in: Cochrane Database Syst Rev.2012;2:CD006697. PMID: 17636847.<br>Reason: Review
55.	Esposito M, Grusovin MG, Tzanetea E, Piattelli A, Worthington HV. Interventions for replacing missing teeth: treatment of perimplantitis. Cochrane Database Syst Rev. 2010 Jun 16;(6):CD004970. doi: 10.1002/14651858.CD004970.pub4. Update in: Cochrane Database Syst Rev. 2012;1:CD004970. PMID: 20556759.<br>Reason: Review
56.	Esposito M, Maghaireh H, Grusovin MG, Ziounas I, Worthington HV. Soft tissue management for dental implants: what are the most effective techniques? A Cochrane systematic review. Eur J Oral Implantol. 2012 Autumn;5(3):221-38. PMID: 23000707.<br>Reason: Review
57.	Esposito M, Maghaireh H, Grusovin MG, Ziounas I, Worthington HV. Interventions for replacing missing teeth: management of soft tissues for dental implants. Cochrane Database Syst Rev. 2012 Feb 15;2012(2):CD006697. doi: 10.1002/14651858.CD006697.pub2. PMID: 22336822; PMCID: PMC6599877.<br>Reason: Review
58.	Faggion CM Jr, Listl S, Frühauf N, Chang HJ, Tu YK. A systematic review and Bayesian network meta-analysis of randomized clinical trials on non-surgical treatments for peri-implantitis. J Clin Periodontol. 2014 Oct;41(10):1015-25. doi: 10.1111/jcpe.12292. Epub 2014 Aug 11. PMID: 25039292.<br>Reason: Review
59.	Farronato D, Mangano F, Briguglio F, Iorio-Siciliano V, Riccitiello F, Guarnieri R. Influence of Laser-Lok surface on immediate functional loading of implants in single-tooth replacement: a 2-year prospective clinical study. Int J Periodontics Restorative Dent. 2014 Jan-Feb;34(1):79-89. doi: 10.11607/prd.1747. PMID: 24396842.<br>Reason: not related to clinical question
60.	Fee LM. Success of surgical interventions for direct dental implant-related injuries to the mandibular nerve: a review. Br J Oral Maxillofac Surg. 2020 Sep;58(7):795-800. doi: 10.1016/j.bjoms.2020.04.012. Epub 2020 May 12. PMID: 32409132.<br>Reason: not related to clinical question
61.	Felice P, Barausse C, Blasone R, Favaretto G, Stacchi C, Calvo M, Marin C, Buti J, Esposito M. A comparison of two dental implant systems in partially edentulous patients: 1-year post-loading results from a pragmatic multicentre randomised controlled trial. Eur J Oral Implantol. 2014 Winter;7(4):397-409. PMID: 25422827.<br>Reason: not related to clinical question
62.	Fröberg KK, Lindh C, Ericsson I. Immediate loading of Brånemark System Implants: a comparison between TiUnite and turned implants placed in the anterior mandible. Clin Implant Dent Relat Res. 2006;8(4):187-97. doi: 10.1111/j.1708-8208.2006.00017.x. PMID: 17100744.<br>Reason: not related to clinical question
63.	Ganeles J, Zöllner A, Jackowski J, ten Bruggenkate C, Beagle J, Guerra F. Immediate and early loading of Straumann implants with a chemically modified surface (SLActive) in the posterior mandible and maxilla: 1-year results from a prospective multicenter study. Clin Oral Implants

	Res. 2008 Nov;19(11):1119-28. doi: 10.1111/j.1600-0501.2008.01626.x. PMID: 18983314.<br>Reason: not related to clinical question
64.	Garbacea A, Lozada JL, Church CA, Al-Ardah AJ, Seiberling KA, Naylor WP, Chen JW. The incidence of maxillary sinus membrane perforation during endoscopically assessed crestal sinus floor elevation: a pilot study. J Oral Implantol. 2012 Aug;38(4):345-59. doi: 10.1563/AAID-JOI-D-12-00083. PMID:22913307.<br>Reason: not related to clinical question
65.	García-Morales JM, Tortamano-Neto P, Todescan FF, de Andrade JC Jr, Marotti J, Zezell DM. Stability of dental implants after irradiation with an 830-nm low-level laser: a double-blind randomized clinical study. Lasers Med Sci. 2012 Jul;27(4):703-11. doi: 10.1007/s10103-011-0948-4. Epub 2011 Jul 6. PMID: 21732113.<br>Reason: non related to clinical question
66.	Geckili O, Bilhan H, Bilgin T. A 24-week prospective study comparing the stability of titanium dioxide grit-blasted dental implants with and without fluoride treatment. Int J Oral Maxillofac Implants. 2009 Jul-Aug;24(4):684-8. PMID: 19885409.<br>Reason: not related to clinical question
67.	Geltzer A, Turalba A, Vedula SS. Surgical implantation of steroids with antiangiogenic characteristics for treating neovascular age-related macular degeneration. Cochrane Database Syst Rev. 2007 Oct 17;(4):CD005022. doi: 0.1002/14651858.CD005022.pub2. Update in: Cochrane Database Syst Rev. 2013;1:CD005022. PMID: 17943833; PMCID: PMC4267224.<br>Reason: not related to clinical question
68.	Gotfredsen K, Karlsson U. A prospective 5-year study of fixed partial prostheses supported by implants with machined and TiO2-blasted surface. J Prosthodont. 2001 Mar;10(1):2-7. doi: 10.1111/j.1532-849x.2001.00002.x. PMID: 11406789.<br>Reason: not related to clinical question
69.	Guarnieri R, Di Nardo D, Di Giorgio G, Miccoli G, Testarelli L. Clinical and radiographics results at 3 years of RCT with split-mouth design of submerged vs. nonsubmerged single laser-microgrooved implants in posterior areas. Int J Implant Dent. 2019 Dec 18;5(1):44. doi: 10.1186/s40729-019-0196-0. PMID:31848762; PMCID: PMC6917687.<br>Reason: not related to clinical question
70.	Guarnieri R, Di Nardo D, Di Giorgio G, Miccoli G, Testarelli L. Influence of the Microgap/Interface Vertical Position on Early Marginal Bone Remodeling Around One-Stage Implants with Laser-Microtextured Collar Surface: A Randomized Clinical Study. Int J Periodontics Restorative Dent. 2019 Jul/Aug;39(4):553-560. doi: 10.11607/prd.3420. PMID: 31226195.<br>Reason: not related to clinical question
71.	Guarnieri R, Grande M, Ippoliti S, Iorio-Siciliano V, Riccitiello F, Farronato D. Influence of a Laser-Lok Surface on Immediate Functional Loading of Implants in Single-Tooth Replacement: Three-Year Results of a Prospective Randomized Clinical Study on Soft Tissue Response and Esthetics. Int J Periodontics Restorative Dent. 2015 Nov-Dec;35(6):865-75. doi: 10.11607/prd.2273. PMID: 26509991.<br>Reason: not related to clinical question
72.	Guarnieri R, Miccoli G, Reda R, Mazzoni A, Di Nardo D, Testarelli L. Sulcus fluid volume, IL-6, and Il-1b concentrations in periodontal and peri-implant tissues comparing machined and laser-microtextured collar/abutment surfaces during 12 weeks of healing: A split-mouth RCT. Clin Oral Implants Res. 2022 Jan;33(1):94-104. doi: 10.1111/clr.13868. Epub 2021 Oct 16. PMID: 34624157.<br>Reason: not related to clinical question
73.	Guarnieri R, Rappelli G, Piemontese M, Procaccini M, Quaranta A. A Double- Blind Randomized Trial Comparing Implants with Laser-Microtextured and Machined Collar Surfaces: Microbiologic and Clinical Results. Int J Oral Maxillofac Implants. 2016 Sep-Oct;31(5):1117-25. doi: 10.11607/jomi.4563. PMID: 27632268.<br>Reason: not related to clinical question

Supplementary TABLE S2

74.	Guo J, Chen X, Xie H, Li T. Efficacy of adjunctive photodynamic therapy to conventional mechanical debridement for peri-implant mucositis. BMC Oral Health. 2024 Apr 16;24(1):464. doi: 10.1186/s12903-024-04198-6. PubMed PMID: 38627721; PubMed Central PMCID: PMC11020816.<br>Reason: Review
75.	Gupta B, Gupta S, Divya D, Dev SV, Bhola S, Guruprasad Y. Comparing the Effectiveness of Different Techniques for the Management of Dental Implant Peri-Implant Mucositis: A Randomized Controlled Trial. J Pharm Bioallied Sci. 2024 Feb;16(Suppl 1):S865-S867. doi: 10.4103/jpbs.jpbs_1063_23. Epub 2024 Feb 29. PubMed PMID: 38595497; PubMed Central PMCID: PMC11001088.<br>Reason: Snot related to clinical question
76.	Haas R, Baron M, Dörtbudak O, Watzek G. Lethal photosensitization, autogenous bone, and e-PTFE membrane for the treatment of peri-implantitis: preliminary results. Int J Oral Maxillofac Implants. 2000 May-Jun;15(3):374-82. PMID: 10874802.<br>Reason: not randomized clinical trial
77.	Hegazy S, Elmekawy N, Emera RM. Peri-implant Outcomes with Laser vs Nanosurface Treatment of Early Loaded Implant-Retaining Mandibular Overdentures. Int J Oral Maxillofac Implants. 2016 Mar-Apr;31(2):424-30. doi:10.11607/jomi.3805. PMID: 27004289.<br>Reason: not related to clinical question
78.	Heintze SD, Zellweger G, Sbicego S, Rousson V, Muñoz-Viveros C, Stober T. Wear of two denture teeth materials in vivo-2-year results. Dent Mater. 2013 Sep;29(9):e191-204. doi: 10.1016/j.dental.2013.04.012. Epub 2013 Jun 6. PMID: 23746749.<br>Reason: not related to clinical question
79.	Hu ML, Zheng G, Lin H, Li N, Zhao PF, Han JM. Network meta-analysis of the treatment efficacy of different lasers for peri-implantitis. Lasers Med Sci. 2021 Apr;36(3):619-629. doi: 10.1007/s10103-020-03101-3. Epub 2021 Feb 16. PMID:33590365.<br>Reason: Review
80.	Huang N, Li Y, Li W, Zhao R, Ou Y, Chen J, Li J. The clinical efficacy of laser in the nonsurgical treatment of peri-implantitis: a systematic review and meta-analysis. Int J Implant Dent. 2024 Nov 14;10(1):54. doi: 10.1186/s40729-024-00570-x. Review. PubMed PMID: 39542954; PubMed Central PMCID: PMC11564455.<br>Reason: Review
81.	Jacobs R, Pittayapat P, van Steenberghe D, De Mars G, Gijbels F, Van Der Donck A, Li L, Liang X, Van Assche N, Quirynen M, Naert I. A split-mouth comparative study up to 16 years of two screw-shaped titanium implant systems. J Clin Periodontol. 2010 Dec;37(12):1119-27. doi: 10.1111/j.1600-051X.2010.01626.x. Epub 2010 Sep 28. PMID: 20874829.<br>Reason: not related to clinical question
82.	Jemt T, Bergendal B, Arvidson K, Bergendal T, Karlsson LD, Linden B, Rundcrantz T, Wendelhag I. Implant-supported welded titanium frameworks in the edentulous maxilla: a 5-year prospective multicenter study. Int J Prosthodont. 2002 Nov-Dec;15(6):544-8. PMID: 12475159.<br>Reason: not related to clinical question
83.	Jemt T, Bergendal B, Arvidsson K, Bergendal T, Karlsson U, Linden B, Palmqvist S, Rundcrantz T, Bergström C. Laser-welded titanium frameworks supported by implants in the edentulous maxilla: a 2-year prospective multicenter study. Int J Prosthodont. 1998 Nov-Dec;11(6):551-7. PMID: 10023217.<br>Reason: not related to clinical question
84.	Jemt T, Henry P, Lindén B, Naert I, Weber H, Bergström C. A comparison of laser-welded titanium and conventional cast frameworks supported by implants in the partially edentulous jaw: a 3-year prospective multicenter study. Int J Prosthodont. 2000 Jul-Aug;13(4):282-8. PMID: 11203643.<br>Reason: not related to clinical question
85.	Jemt T, Henry P, Lindén B, Naert I, Weber H, Wendelhag I. Implant-supported laser-welded titanium and conventional cast frameworks in the partially edentulous law: a 5-year

	prospective multicenter study. Int J Prosthodont. 2003 Jul-Aug;16(4):415-21. PMID: 12956498.<br>Reason: not related to clinical question
86.	Jervøe-Storm PM, Bunke J, Worthington HV, Needleman I, Cosgarea R, MacDonald L, Walsh T, Lewis SR, Jepsen S. Adjunctive antimicrobial photodynamic therapy for treating periodontal and peri-implant diseases. Cochrane Database Syst Rev. 2024 Jul 12;7(7):CD011778. doi: 10.1002/14651858.CD011778.pub2. Review. PubMed PMID: 38994711; PubMed Central PMCID: PMC11240860.<br>Reason: Review
87.	Kanao M, Nakamoto T, Kajiwara N, Kondo Y, Masaki C, Hosokawa R. Comparison of plaque accumulation and soft-tissue blood flow with the use of full-arch implant-supported fixed prostheses with mucosal surfaces of different materials: a randomized clinical study. Clin Oral Implants Res. 2013 Oct;24(10):1137-43. doi: 10.1111/j.1600-0501.2012.02523.x. Epub 2012 Jul 18. PMID: 22804816.<br>Reason: not related to clinical question
88.	Kaner D, Zhao H, Terheyden H, Friedmann A. Improvement of microcirculation and wound healing in vertical ridge augmentation after pre-treatment with self- inflating soft tissue expanders - a randomized study in dogs. Clin Oral Implants Res. 2015 Jun;26(6):720-4. doi: 10.1111/clr.12377. Epub 2014 Apr 16. PMID: 24735431.<br>Reason: not related to clinical question
89.	Karimi MR, Farkhondemehr B, Ghaeni Najafi M, Etemadi A, Chiniforush N. Efficacy of titanium brush, 915 nm diode laser, citric acid for eradication of Staphylococcus aureus from implant surfaces. BMC Oral Health. 2021 Dec 7;21(1):631. doi: 10.1186/s12903-021-01997-z. PMID: 34876098; PMCID: PMC8650515.<br>Reason: in vitro study
90.	Karimi MR, Hasani A, Khosroshahian S. Efficacy of Antimicrobial Photodynamic Therapy as an Adjunctive to Mechanical Debridement in the Treatment of Peri-implant Diseases: A Randomized Controlled Clinical Trial. J Lasers Med Sci. 2016 Summer;7(3):139-145. doi: 10.15171/jlms.2016.24. Epub 2016 Jul 18. PMID: 28144432; PMCID: PMC5262478.<br>Reason: insufficient follow-up
91.	Kasperski J, Rosak P, Rój R, Czelakowska A, Sieroń-Stołtny K, Pasek J, Sieroń A. The influence of low-frequency variable magnetic fields in reducing pain experience after dental implant treatment. Acta Bioeng Biomech. 2015;17(4):97-105. PMID: 26898264.<br>Reason: not related to clinical question
92.	Keeve PL, Koo KT, Ramanauskaite A, Romanos G, Schwarz F, Sculean A, Khoury F. Surgical Treatment of Periimplantitis With Non-Augmentative Techniques. Implant Dent. 2019 Apr;28(2):177-186. doi: 10.1097/ID.0000000000000838. PMID: 30475243.<br>Reason: Not related to the clinical question
93.	Kinalski MA, Agostini BA, Bergoli CD, Dos Santos MBF. Influence of low-level laser therapy on implant stability in implants placed in healed sites: a randomized controlled trial. Int J Implant Dent. 2021 Jun 1;7(1):49. doi: 10.1186/s40729-021-00331-0. PMID: 34059974; PMCID: PMC8166992.<br>Reason: not related to clinical question
94.	Koh RU, Oh TJ, Rudek I, Neiva GF, Misch CE, Rothman ED, Wang HL. Hard and soft tissue changes after crestal and subcrestal immediate implant placement. J Periodontol. 2011 Aug;82(8):1112-20. doi: 10.1902/jop.2011.100541. Epub 2011 Feb 2. PMID: 21284552.<br>Reason: not related to clinical question
95.	Kulshrestha RS, Tandon R, Chandra P. Canine retraction: A systematic review of different methods used. J Orthod Sci. 2015 Jan-Mar;4(1):1-8. doi: 10.4103/2278-0203.149608. PMID: 25657985; PMCID: PMC4314834.<br>Reason: not related to clinical question
96.	Kuzu TE, Öztürk K, Gürgan CA, Üşümez A, Yay A, Göktepe Ö. Effect of Photobiomodulation Therapy on Peri-Implant Bone Healing in Extra-Short Implants in a Rabbit Model: A Pilot Study. Photobiomodul Photomed Laser Surg. 2022 Jun;40(6):402-409. doi:

	10.1089/photob.2021.0098. PMID: 35749706.<br>Reason: not related to clinical question
97.	Leretter M, Cândea A., Topala L. Photodynamic Therapy in Peri-implantitis. Downloaded From: http://proceedings.spiedigitallibrary.org/ on 05/12/2015 Terms of Use: http://spiedl.org/terms<br>Reason: congress proceedings
98.	Liddelow G, Henry P. The immediately loaded single implant-retained mandibular overdenture: a 36-month prospective study. Int J Prosthodont. 2010 Jan-Feb;23(1):13-21. PMID: 20234886.<br>Reason: not related to clinical question
99.	Lin GH, Suárez López Del Amo F, Wang HL. Laser therapy for treatment of peri- implant mucositis and peri-implantitis: An American Academy of Periodontology best evidence review. J Periodontol. 2018 Jul;89(7):766-782. doi: 10.1902/jop.2017.160483. PMID: 30133748.<br>Reason: Review
100.	Liu S, Wang B, Chen Z, Mo H, Lin M, Huang X. Meta-analysis of the Efficacy of Photodynamic Therapy (PDT) in the Treatment of Peri-implantitis. Altern Ther Health Med. 2024 Nov;30(11):312-318. PubMed PMID: 38687855.<br>Reason: Review
101.	Lobato RPB, Kinalski MA, Martins TM, Agostini BA, Bergoli CD, Dos Santos MBF. Influence of low-level laser therapy on implant stability in implants placed in fresh extraction sockets: A randomized clinical trial. Clin Implant Dent Relat Res. 2020 Jun;22(3):261-269. doi: 10.1111/cid.12904. Epub 2020 Apr 24. PMID: 32329198.<br>Reason: not related to clinical question
102.	Lowy J, Kwon HS, Patel A, Greenwell H, Hill M, Katwal D, Rademacher AC, Mendoza J. The Effect of Platform-Switching Plus Laser Grooving on Peri-implant Hard and Soft Tissue Level: A Randomized, Controlled, Blinded Clinical Trial. Int J Periodontics Restorative Dent. 2019 Sep/Oct;39(5):669-674. doi: 10.11607/prd.4243. PMID: 31449577.<br>Reason: not related to clinical question
103.	Malmqvist S, Qadri T, Lira-Junior R, Boström EA, Gustafsson A, Belibasakis GN, Silbereisen A, Johannsen G, Johannsen A. Treatment of peri-implantitis with diode laser or mucosal flap surgery: A clinical randomized controlled trial. J Periodontol. 2025 Mar 24;. doi: 10.1002/JPER.24-0683. [Epub ahead of print] PubMed PMID: 40125562.<br>Reason: Studies utilizing low level laser therapy or laser therapy alone
104.	Mandić B, Lazić Z, Marković A, Mandić B, Mandić M, Djinić A, Miličić B. Influence of postoperative low-level laser therapy on the osseointegration of self-tapping implants in the posterior maxilla: a 6-week split-mouth clinical study. Vojnosanit Pregl. 2015 Mar;72(3):233-40. doi: 10.2298/vsp131202075m. PMID: 25958474.<br>Reason: not related to clinical question
105.	Mangano C, Mangano FG, Shibli JA, Roth LA, d' Addazio G, Piattelli A, Iezzi G. Immunohistochemical Evaluation of Peri-Implant Soft Tissues around Machined and Direct Metal Laser Sintered (DMLS) Healing Abutments in Humans. Int J Environ Res Public Health. 2018 Jul 30;15(8):1611. doi: 10.3390/ijerph15081611. PMID: 30061523; PMCID: PMC6121298.<br>Reason: not related to clinical question
106.	Mariani GM, Ercoli E, Guzzi N, Bongiovanni L, Bianco L, Romano F, Aimetti M. One-year clinical outcomes following non-surgical treatment of peri-implant mucositis with adjunctive diode laser application. Minerva Stomatol. 2020 Oct;69(5):269-277. doi: 10.23736/S0026-4970.20.04340-X. Epub 2020 Apr 10. PMID: 32278340.<br>Reason: Studies utilizing low level laser therapy or laser therapy alone
107.	Martellacci L, Quaranta G, Patini R, Isola G, Gallenzi P, Masucci L. A Literature Review of Metagenomics and Culturomics of the Peri-implant Microbiome: Current Evidence and Future Perspectives. Materials (Basel). 2019 Sep 17;12(18):3010. doi: 10.3390/ma12183010. PMID: 31533226; PMCID: PMC6766346.<br>Reason: Review
108.	Matys J, Świder K, Grzech-Leśniak K, Dominiak M, Romeo U. Photobiomodulation by a 635nm Diode Laser on Peri-Implant Bone: Primary and Secondary Stability and Bone Density Analysis-A

	Randomized Clinical Trial. Biomed Res Int. 2019 Apr 22;2019:2785302. doi: 10.1155/2019/2785302. PMID: 31143771; PMCID: PMC6501257.<br>Reason: not related to clinical question
109.	Maurya RK, Gupta A, Singh H, Thakkar S, Mishra HA. Effects of Photodynamic Therapy on the Clinical and Biomechanical Efficiency of Mini-Implants: A Randomized Controlled Trial. J Clin Orthod. 2017 May;51(5):259-269. PMID: 28668941.<br>Reason: not related to clinical question
110.	Menicucci G, Mussano F, Schierano G, Rizzati A, Aimetti M, Gassino G, Traini T, Carossa S. Healing properties of implants inserted concomitantly with anorganic bovine bone. A histomorphometric human study. Aust Dent J. 2013 Mar;58(1):57-66. doi: 10.1111/adj.12032. Epub 2013 Feb 5. PMID: 23441793.<br>Reason: not related to clinical question
111.	Mizutani K, Aoki A, Coluzzi D, Yukna R, Wang CY, Pavlic V, Izumi Y. Lasers in minimally invasive periodontal and peri-implant therapy. Periodontol 2000. 2016 Jun;71(1):185-212. doi: 10.1111/prd.12123. PMID: 27045437.<br>Reason: Studies utilizing low level laser therapy or laser therapy alone
112.	Monea A, Beresescu G, Boeriu S, Tibor M, Popsor S, Antonescu DM. Bone healing after low-level laser application in extraction sockets grafted with allograft material and covered with a resorbable collagen dressing: a pilot histological evaluation. BMC Oral Health. 2015 Oct 29;15:134. doi:10.1186/s12903-015-0122-7. Erratum in: BMC Oral Health. 2016;16:16. Boeriu,Sorin [added]. PMID: 26511218; PMCID: PMC4625559.<br>Reason: not related to clinical question
113.	Mongardini C, Pilloni A, Farina R, Di Tanna G, Zeza B. Adjunctive efficacy of probiotics in the treatment of experimental peri-implant mucositis with mechanical and photodynamic therapy: a randomized, cross-over clinical trial. J Clin Periodontol. 2017 Apr;44(4):410-417. doi: 10.1111/jcpe.12689. Epub 2017 Feb 18. PMID: 28032908.<br>Reason: Studies using additional antibiotic or other adjunctive therapy in the test or control group
114.	Munhoz EA, Bodanezi A, Cestari Biol TM, Zardin Graeff MS, Junior OF, de Carvalho PSP, Taga R. Impact of Inorganic Xenograft on Bone Healing and Osseointegration: An Experimental Study in Rabbits. Implant Dent. 2017 Dec;26(6):875-881. doi: 10.1097/ID.0000000000000694. PMID: 29095789.<br>Reason: not related to clinical question
115.	Murakami-Malaquias-Silva F, Rosa EP, Almeida PA, Schalch TO, Tenis CA, Negreiros RM, Horliana RF, Garcez AS, Fernandes MUR, Tortamano A, Motta LJ, Bussadori SK, Horliana ACRT. Evaluation of the effects of photobiomodulation on orthodontic movement of molar verticalization with mini-implant: A randomized double-blind protocol study. Medicine (Baltimore). 2020 Mar;99(13):e19430. doi: 10.1097/MD.0000000000019430. PMID: 32221067; PMCID: PMC7220149.<br>Reason: not related to clinical question
116.	Muzaheed, Acharya S, Hakami AR, Allemailem KS, Alqahtani K, Al Saffan A, Aldakheel FM, Divakar DD. Effectiveness of single versus multiple sessions of photodynamic therapy as adjunct to scaling and root planing on periodontopathogenic bacteria in patients with periodontitis. Photodiagnosis Photodyn Ther. 2020 Dec;32:102035. doi: 10.1016/j.pdpdt.2020.102035. Epub 2020 Oct 2. PMID: 33011399.<br>Reason: not related to clinical question
117.	Nejat AH, Eshghpour M, Danaeifar N, Abrishami M, Vahdatinia F, Fekrazad R. Effect of Photobiomodulation on the Incidence of Alveolar Osteitis and Postoperative Pain following Mandibular Third Molar Surgery: A Double-Blind Randomized Clinical Trial. Photochem Photobiol. 2021 Sep;97(5):1129-1135. doi: 10.1111/php.13457. Epub 2021 Jun 17. PMID: 34048061.<br>Reason: not related to clinical question

118.	Nuñez-Pantoja JM, Vaz LG, Nóbilo MA, Henriques GE, Mesquita MF. Effects of laser-weld joint opening size on fatigue strength of Ti-6Al-4V structures with several diameters. J Oral Rehabil. 2011 Mar;38(3):196-201. doi: 10.1111/j.1365-2842.2010.02140.x. PMID: 20678101.<br>Reason: not related to clinical question
119.	Ohba S, Sato M, Noda S, Yamamoto H, Egahira K, Asahina I. Assessment of safety and efficacy of antimicrobial photodynamic therapy for peri-implant disease. Photodiagnosis Photodyn Ther. 2020 Sep;31:101936. doi: 10.1016/j.pdpdt.2020.101936. Epub 2020 Aug 11. PMID: 32791295.<br>Reason: insufficient follow-up
120.	Orsini G, Piattelli M, Scarano A, Petrone G, Kenealy J, Piattelli A, Caputi S. Randomized, controlled histologic and histomorphometric evaluation of implants with nanometer-scale calcium phosphate added to the dual acid-etched surface in the human posterior maxilla. J Periodontol. 2007 Feb;78(2):209-18. doi: 10.1902/jop.2007.060297. PMID: 17274708.<br>Reason: not related to clinical question
121.	Ortorp A, Jemt T. Clinical experiences with laser-welded titanium frameworks supported by implants in the edentulous mandible: a 10-year follow-up study. Clin Implant Dent Relat Res. 2006;8(4):198-209. doi: 10.1111/j.1708-8208.2006.00020.x. PMID: 17100745.<br>Reason: not related to clinical question
122.	Ortorp A, Jemt T. Early laser-welded titanium frameworks supported by implants in the edentulous mandible: a 15-year comparative follow-up study. Clin Implant Dent Relat Res. 2009 Dec;11(4):311-22. doi:10.1111/j.1708-8208.2008.00119.x. Epub 2008 Sep 9. PMID: 18783415.<br>Reason: not related to clinical question
123.	Ortorp A, Jemt T. Laser-welded titanium frameworks supported by implants in the partially edentulous mandible: a 10-year comparative follow-up study. Clin Implant Dent Relat Res. 2008 Sep;10(3):128-39. doi: 10.1111/j.1708-8208.2007.00073.x. Epub 2008 Jan 24. PMID: 18218053.<br>Reason: not related to clinical question
124.	Ortorp A, Linden B, Jemt T. Clinical experiences with laser-welded titanium frameworks supported by implants in the edentulous mandible: a 5-year follow-up study. Int J Prosthodont. 1999 Jan-Feb;12(1):65-72. PMID: 10196830.<br>Reason: not related to clinical question
125.	Ower PC, Ciantar M, Newman HN, Wilson M, Bulman JS. The effects on chronic periodontitis of a subgingivally-placed redox agent in a slow release device. J Clin Periodontol. 1995 Jun;22(6):494-500. doi: 10.1111/j.1600-051x.1995.tb00184.x. PMID: 7560231.<br>Reason: not related to clinical question
126.	Oyagüe RC, Sánchez-Turrión A, López-Lozano JF, Montero J, Albaladejo A, Suárez-García MJ. Evaluation of fit of cement-retained implant-supported 3-unit structures fabricated with direct metal laser sintering and vacuum casting techniques. Odontology. 2012 Jul;100(2):249-53. doi: 10.1007/s10266-011-0050-1. Epub 2011 Nov 11. PMID: 22075754.<br>Reason: not related to clinical question
127.	Paiva J, Givan DA, Broome JC, Lemons JE, McCracken MS. Comparison of the passivity between cast alloy and laser-welded titanium overdenture bars. J Prosthodont. 2009 Dec;18(8):656-62. doi: 10.1111/j.1532-849X.2009.00504.x. Epub 2009 Aug 13. PMID: 19682220.<br>Reason: not related to clinical question
128.	Palled V, Rao J, Singh RD, Tripathi S, Singh K, Radav R, Verma U, Chand P. Assessment of the Healing of Dental Implant Surgical Site Following Low-Level Laser Therapy Using Bioclinical Parameters: An Exploratory Study. J Oral Implantol. 2021 Jun 1;47(3):230-235. doi: 10.1563/aaid-joi-D-18-00316. PMID: 32662840.<br>Reason: not related to clinical question
129.	Paolantonio M, Dolci M, Scarano A, d'Archivio D, di Placido G, Tumini V, Piattelli A. Immediate implantation in fresh extraction sockets. A controlled clinical and histological study in man. J Periodontol. 2001 Nov;72(11):1560-71. doi: 10.1902/jop.2001.72.11.1560. PMID: 11759868.<br>Reason: not related to clinical question

Supplementary TABLE S2

130.	Papadopoulos CA, Vouros I, Menexes G, Konstantinidis A. The utilization of a diode laser in the surgical treatment of peri-implantitis. A randomized clinical trial. Clin Oral Investig. 2015 Nov;19(8):1851-60. doi: 10.1007/s00784-014-1397-9. Epub 2015 Jan 28. PMID: 25623382.<br>Reason: Studies utilizing low level laser therapy or laser therapy alone
131.	Peng X, Guo X, Zhou Y. The Efficacy of Ten Different Adjunctive Measures in Patients with Nonsurgically Treated Peri-Implant Disease: A Network Meta-Analysis of Randomized Controlled Trials. Photobiomodul Photomed Laser Surg. 2024 Feb;42(2):99-124. doi: 10.1089/photob.2023.0109. Epub 2024 Jan 31. Review. PubMed PMID: 38294889.<br>Reason: Review
132.	Persson GR, Roos-Jansåker AM, Lindahl C, Renvert S. Microbiologic results after non-surgical erbium-doped:yttrium, aluminum, and garnet laser or air- abrasive treatment of peri-implantitis: a randomized clinical trial. J Periodontol. 2011 Sep;82(9):1267-78. doi: 10.1902/jop.2011.100660. Epub 2011 Mar 21. PMID: 21417591.<br>Reason: Studies utilizing low level laser therapy or laser therapy alone
133.	Pourabbas R, Khorramdel A, Sadighi M, Kashefimehr A, Mousavi SA. Effect of photodynamic therapy as an adjunctive to mechanical debridement on the nonsurgical treatment of peri-implant mucositis: A randomized controlled clinical trial. Dent Res J (Isfahan). 2023;20:1. eCollection 2023. PubMed PMID: 36820137; PubMed Central PMCID: PMC9937927.<br>Reason: Insufficient follow-up
134.	Rakašević D, Lazić Z, Rakonjac B, Soldatović I, Janković S, Magić M, Aleksić Z. Efficiency of photodynamic therapy in the treatment of peri-implantitis – A three-month randomized controlled clinical trial. Srp Arh Celok Lek. 2016 Sep- Oct;144(9-10):478-84. PMID: 29652462.<br>Reason: insufficient follow-up
135.	Rakasevic D. ; Z. Lazic; Z. Aleksic; S. Jankovic; Natasa Jakoba Nikolic 1 ; Ivan Soldatovic 3 ; Ljiljana Djukic. Evaluation of clinical and immunological parameters after applying the adjunctive photodynamic therapy in the surgical treatment of peri- implantitis. A 6- and 12-month randomized controlled clinical trial. Oral Communication Clinical ReseArch Perimplant Biology 2019.<br>Reason: congress proceedings
136.	Rakasevic D.L., Z. Lazic, S.M. Jankovic, N. Nikolic Jakoba, I. Soldatovic, J. Roganovic, Z.M. Aleksic. Clinical and immunological response to photodynamic therapy in the treatment of peri-implantitis. Oral Presentation. 2018 European Federation of Periodontology.<br>Reason: congress proceedings
137.	Reith S, Radke PW, Volk O, vom Dahl J, Klues HG. The place of rotablator for treatment of in-stent restenosis. Semin Interv Cardiol. 2000 Dec;5(4):199-208. doi: 10.1053/siic.2000.0140. PMID: 11244517.<br>Reason: not related to clinical question
138.	Renvert S, Lindahl C, Roos Jansåker AM, Persson GR. Treatment of peri-implantitis using an Er:YAG laser or an air-abrasive device: a randomized clinical trial. J Clin Periodontol. 2011 Jan;38(1):65-73. doi: 10.1111/j.1600-051X.2010.01646.x. Epub 2010 Nov 22. PMID: 21091527.<br>Reason: Studies utilizing low level laser therapy or laser therapy alone
139.	Renvert S, Roos-Jansåker AM, Claffey N. Non-surgical treatment of peri-implant mucositis and peri-implantitis: a literature review. J Clin Periodontol. 2008 Sep;35(8 Suppl):305-15. doi: 10.1111/j.1600-051X.2008.01276.x. PMID: 18724858.<br>Reason: Review
140.	Roccuzzo A, Klossner S, Stähli A, Imber JC, Eick S, Sculean A, Salvi GE. Non-surgical mechanical therapy of peri-implantitis with or without repeated adjunctive diode laser application. A 6-month double-blinded randomized clinical trial. Clin Oral Implants Res. 2022 Sep;33(9):900-912. doi: 10.1111/clr.13969. Epub 2022 Jul 10. PMID: 35775311; PMCID: PMC9546299.<br>Reason: Studies utilizing low level laser therapy or laser therapy alone
141.	Romão MM, Marques MM, Cortes AR, Horliana AC, Moreira MS, Lascala CA. Micro- computed tomography and histomorphometric analysis of human alveolar bone repair induced by laser

	phototherapy: a pilot study. Int J Oral Maxillofac Surg. 2015 Dec;44(12):1521-8. doi: 10.1016/j.ijom.2015.08.989. Epub 2015 Sep 14. PMID:26381208.<br>Reason: not related to clinical question
142.	Ryan LL, Kohles SS. A temporospatial histomorphometric analysis of bone density adjacent to acid-etched self-tapping dental implants with an external hexagon connection in the female baboon. Clin Oral Investig. 2022 Feb;26(2):2143-2154. doi: 10.1007/s00784-021-04195-x. Epub 2021 Sep 29. PMID: 34585261.<br>Reason: not related to clinical question
143.	Sabuncuoglu FA, Ersahan S. Changes in maxillary incisor dental pulp blood flow during intrusion by mini-implants. Acta Odontol Scand. 2014 Oct;72(7):489-96. doi: 10.3109/00016357.2013.867362. Epub 2014 Jan 20. PMID: 24438561.<br>Reason: not related to clinical question
144.	Sabuncuoglu FA, Ersahan S. Comparative evaluation of pulpal blood flow during incisor intrusion. Aust Orthod J. 2015 Nov;31(2):171-7. PMID: 26999890.<br>Reason: not related to clinical question
145.	Sánchez-Martos R, Samman A, Priami M, Arias-Herrera S. The diode laser as coadyuvant therapy in the non-surgical conventional treatment of peri-implant mucositis: A systematic review and meta-analysis. J Clin Exp Dent. 2020 Dec 1;12(12):e1171-e1182. doi: 10.4317/jced.57630. PMID: 33282139; PMCID: PMC7700786.<br>Reason: Review
146.	Sayardoust S, Omar O, Norderyd O, Thomsen P. Clinical, radiological, and gene expression analyses in smokers and non-smokers, Part 2: RCT on the late healing phase of osseointegration. Clin Implant Dent Relat Res. 2017 Oct;19(5):901-915. doi: 10.1111/cid.12514. Epub 2017 Jul 26. PMID: 28744993.<br>Reason: not related to clinical question
147.	Schär D, Ramseier CA, Eick S, Arweiler NB, Sculean A, Salvi GE. Anti- infective therapy of peri-implantitis with adjunctive local drug delivery or photodynamic therapy: six-month outcomes of a prospective randomized clinical trial. Clin Oral Implants Res. 2013 Jan;24(1):104-10. doi:10.1111/j.1600-0501.2012.02494.x. Epub 2012 May 9. PMID: 22568744.<br>Reason: Studies using additional antibiotic or other adjunctive therapy in the test or control group
148.	Schincaglia GP, Marzola R, Scapoli C, Scotti R. Immediate loading of dental implants supporting fixed partial dentures in the posterior mandible: a randomized controlled split-mouth study--machined versus titanium oxide implant surface. Int J Oral Maxillofac Implants. 2007 Jan-Feb;22(1):35-46. PMID: 17340895.<br>Reason: not related to clinical question
149.	Schwarz F, Bieling K, Bonsmann M, Latz T, Becker J. Nonsurgical treatment of moderate and advanced periimplantitis lesions: a controlled clinical study. Clin Oral Investig. 2006 Dec;10(4):279-88. doi: 10.1007/s00784-006-0070-3. Epub 2006 Sep 13. PMID: 16969659.<br>Reason: Studies utilizing low level laser therapy or laser therapy alone
150.	Schwarz F, Bieling K, Nuesry E, Sculean A, Becker J. Clinical and histological healing pattern of peri-implantitis lesions following non-surgical treatment with an Er:YAG laser. Lasers Surg Med. 2006 Aug;38(7):663-71. doi: 10.1002/lsm.20347. PMID: 16634072.<br>Reason: Studies using additional antibiotic or other adjunctive therapy in the test or control group
151.	Schwarz F, Bieling K, Sculean A, Herten M, Becker J. Laser und Ultraschall in der Therapie periimplantärer Infektionen--eine Literaturübersicht [Treatment of periimplantitis with laser or ultrasound. A review of the literature]. Schweiz Monatsschr Zahnmed. 2004;114(12):1228-35. German. PMID: 15646670.<br>Reason: Review
152.	Schwarz F, Hegewald A, John G, Sahm N, Becker J. Four-year follow-up of combined surgical therapy of advanced peri-implantitis evaluating two methods of surface decontamination. J Clin Periodontol. 2013 Oct;40(10):962-7. doi: 10.1111/jcpe.12143. Epub 2013 Aug 12. PMID:

	23931259.<br>Reason: Studies utilizing low level laser therapy or laser therapy alone
153.	Schwarz F, John G, Mainusch S, Sahm N, Becker J. Combined surgical therapy of peri-implantitis evaluating two methods of surface debridement and decontamination. A two-year clinical follow up report. J Clin Periodontol. 2012 Aug;39(8):789-97. doi: 10.1111/j.1600-051X.2012.01867.x. Epub 2012 May 28. PMID:22639800.<br>Reason: not related to clinical question
154.	Schwarz F, Sahm N, Iglhaut G, Becker J. Impact of the method of surface debridement and decontamination on the clinical outcome following combined surgical therapy of peri-implantitis: a randomized controlled clinical study. J Clin Periodontol. 2011 Mar;38(3):276-84. doi:10.1111/j.1600-051X.2010.01690.x. Epub 2011 Jan 11. PMID: 21219392.<br>Reason: Studies utilizing low level laser therapy or laser therapy alone
155.	Schwarz F, Sculean A, Romanos G, Herten M, Horn N, Scherbaum W, Becker J. Influence of different treatment approaches on the removal of early plaque biofilms and the viability of SAOS2 osteoblasts grown on titanium implants. Clin Oral Investig. 2005 Jun;9(2):111-7. doi: 10.1007/s00784-005-0305-8. Epub 2005 Apr 20. PMID: 15841403.<br>Reason: not related to clinical question
156.	Schwarz F, Sculean A, Rothamel D, Schwenzer K, Georg T, Becker J. Clinical evaluation of an Er:YAG laser for nonsurgical treatment of peri-implantitis: a pilot study. Clin Oral Implants Res. 2005 Feb;16(1):44-52. doi: 10.1111/j.1600-0501.2004.01051.x. PMID: 15642030.<br>Reason: Studies utilizing low level laser therapy or laser therapy alone
157.	Sennhenn-Kirchner S, Klaue S, Wolff N, Mergeryan H, Borg von Zepelin M, Jacobs HG. Decontamination of rough titanium surfaces with diode lasers: microbiological findings on in vivo grown biofilms. Clin Oral Implants Res. 2007 Feb;18(1):126-32. doi: 10.1111/j.1600-0501.2006.01298.x. PMID: 17224033.<br>Reason: in vitro study
158.	Shahnaz A, Jamali R, Mohammadi F, Khorsand A, Moslemi N, Fekrazad R. A preliminary randomized clinical trial comparing diode laser and scalpel periosteal incision during implant surgery: impact on postoperative morbidity and implant survival. Lasers Med Sci. 2018 Jan;33(1):19-25. doi:10.1007/s10103-017-2315-6. Epub 2017 Aug 31. PMID: 28861729.<br>Reason: not related to clinical question
159.	Shetty B, Ali D, Ahmed S, et al. Role of antimicrobial photodynamic therapy in reducing subgingival oral yeasts colonization in patients with peri-implant mucositis. Photodiagnosis Photodyn Ther. 2022;38:102803. doi:10.1016/j.pdpdt.2022.102803<br>Reason: insufficient follow-up
160.	Shetty B, Ali D, Ahmed S, Ibraheem WI, Preethanath RS, Vellappally S, Divakar DD. Role of antimicrobial photodynamic therapy in reducing subgingival oral yeasts colonization in patients with peri-implant mucositis. Photodiagnosis Photodyn Ther. 2022 Jun;38:102803. doi: 10.1016/j.pdpdt.2022.102803. Epub 2022 Mar 11. PMID: 35288320.<br>Reason: insufficient follow-up
161.	Sivaramakrishnan G, Sridharan K. Photodynamic therapy for the treatment of peri-implant diseases: A network meta-analysis of randomized controlled trials. Photodiagnosis Photodyn Ther. 2018 Mar;21:1-9. doi: 10.1016/j.pdpdt.2017.10.013. Epub 2017 Oct 24. PMID: 29079351.<br>Reason: Review
162.	Srinivasan M, Kamnoedboon P, Papi P, Romeo U. Efficacy of non-surgical laser therapy for the management of peri-implantitis: A systematic review and meta-analysis. J Dent. 2025 Mar;154:105562. doi: 10.1016/j.jdent.2025.105562. Epub 2025 Jan 11. Review. PubMed PMID: 39805490.<br>Reason: Review
163.	Strauss G, Goteiner D, Murawski K, Singer SR, Drew HJ, Sullivan A. Laser-Assisted Therapy for the Treatment of Peri-implantitis. Part I. Clinical Outcomes. Int J Periodontics Restorative Dent. 2021 Jul-Aug;41(4):563-568. doi:10.11607/prd.5377. PMID: 34328476.<br>Reason: Studies utilizing low level laser therapy or laser therapy alone

164.	Tenore G, Montori A, Mohsen A, Mattarelli G, Palaia G, Romeo U. Evaluation of adjunctive efficacy of diode laser in the treatment of peri-implant mucositis: a randomized clinical trial. Lasers Med Sci. 2020 Aug;35(6):1411-1417. doi: 10.1007/s10103-020-03009-y. Epub 2020 Apr 18. PMID:32304001.<br>Reason: Studies utilizing low level laser therapy or laser therapy alone
165.	Torkzaban P, Kasraei S, Torabi S, Farhadian M. Low-level laser therapy with 940 nm diode laser on stability of dental implants: a randomized controlled clinical trial. Lasers Med Sci. 2018 Feb;33(2):287-293. doi: 10.1007/s10103-017-2365-9. Epub 2017 Oct 29. PMID: 29082462.<br>Reason: not related to clinical question
166.	Van Steenberghe D, De Mars G, Quirynen M, Jacobs R, Naert I. A prospective split-mouth comparative study of two screw-shaped self-tapping pure titanium implant systems. Clin Oral Implants Res. 2000 Jun;11(3):202-9. doi: 10.1034/j.1600-0501.2000.011003202.x. PMID: 11168211.<br>Reason: not related to clinical question
167.	Verdonck HW, Meijer GJ, Kessler P, Nieman FH, de Baat C, Stoelinga PJ. Assessment of bone vascularity in the anterior mandible using laser Doppler flowmetry. Clin Oral Implants Res. 2009 Feb;20(2):140-4. doi: 10.1111/j.1600-0501.2008.01631.x. Epub 2008 Dec 1. PMID: 19077149.<br>Reason: not related to clinical question
168.	Verket A, Koldsland OC, Bunaes D, Lie SA, Romandini M. Non-surgical therapy of peri-implant mucositis-Mechanical/physical approaches: A systematic review. J Clin Periodontol. 2023 Jun;50 Suppl 26:135-145. doi: 10.1111/jcpe.13789. Epub 2023 Feb 28. Review. PubMed PMID: 36802083.<br>Reason: Review
169.	Wang CW, Ashnagar S, Gianfilippo RD, Arnett M, Kinney J, Wang HL. Laser-assisted regenerative surgical therapy for peri-implantitis: A randomized controlled clinical trial. J Periodontol. 2021 Mar;92(3):378-388. doi: 10.1002/JPER.20-0040. Epub 2020 Aug 25. PMID: 32761810.<br>Reason: Studies utilizing low level laser therapy or laser therapy alone
170.	Wennerberg A, Fröjd V, Olsson M, Nannmark U, Emanuelsson L, Johansson P, Josefsson Y, Kangasniemi I, Peltola T, Tirri T, Pänkäläinen T, Thomsen P. Nanoporous TiO(2) thin film on titanium oral implants for enhanced human soft tissue adhesion: a light and electron microscopy study. Clin Implant Dent Relat Res. 2011 Sep;13(3):184-96. doi: 10.1111/j.1708-8208.2009.00207.x. Epub 2009 Aug 3. PMID: 19681943.<br>Reason: not related to clinical question
171.	Yan M, Liu M, Wang M, Yin F, Xia H. The effects of Er:YAG on the treatment of peri-implantitis: a meta-analysis of randomized controlled trials. Lasers Med Sci. 2015 Sep;30(7):1843-53. doi: 10.1007/s10103-014-1692-3. Epub 2014 Nov 27. PMID: 25428598.<br>Reason: Review
172.	Zadeh HH, Abdelhamid A, Omran M, Bakhshalian N, Tarnow D. An open randomized controlled clinical trial to evaluate ridge preservation and repair using SocketKAP(™) and SocketKAGE(™) : part 1-three-dimensional volumetric soft tissue analysis of study casts. Clin Oral Implants Res. 2016 Jun;27(6):640-9. doi: 10.1111/clr.12714. Epub 2015 Nov 2. PMID: 26521754.<br>Reason: not related to clinical question
173.	Zeza B, Farina R, Pilloni A, Mongardini C. Clinical outcomes of experimental gingivitis and peri-implant mucositis treatment with professionally administered plaque removal and photodynamic therapy. Int J Dent Hyg. 2018 May;16(2):e58-e64. doi: 10.1111/idh.12302. Epub 2017 Aug 11. PMID: 28799232.<br>Reason: insufficient follow-up
174.	Zipprich H, Weigl P, Di Gianfilippo R, Steigmann L, Henrich D, Wang HL, Schlee M, Ratka C. Comparison of decontamination efficacy of two electrolyte cleaning methods to diode laser, plasma, and air-abrasive devices. Clin Oral Investig. 2022 Jun;26(6):4549-4558. doi: 10.1007/s00784-022-04421-0. Epub 2022 Mar 24. PMID: 35322316.<br>Reason: in vitro study

175.	Zöllner A, Ganeles J, Korostoff J, Guerra F, Krafft T, Brägger U. Immediate and early non-occlusal loading of Straumann implants with a chemically modified surface (SLActive) in the posterior mandible and maxilla: interim results from a prospective multicenter randomized-controlled study. Clin Oral Implants Res. 2008 May;19(5):442-50. doi: 10.1111/j.1600-0501.2007.01517.x. PMID: 18416725.<br>Reason: not related to clinical question
